# Supplementary material for: One substrate many enzymes virtual screening uncovers missing genes of carnitine biosynthesis in human and mouse
Source: Nat Commun. 2024 Apr 13;15:3199. doi: 10.1038/s41467-024-47466-3 (PMC11016064; doi:10.1038/s41467-024-47466-3)
Supplement: Supplementary file 1 — Supplementary Information [file 41467_2024_47466_MOESM1_ESM.pdf]

Supplementary Information for

One substrate many enzymes virtual screening uncovers missing genes of carnitine biosynthesis in human and mouse

Marco Malatesta[1], Emanuele Fornasier[2], Martino Luigi Di Salvo[3], Angela Tramonti[4], Erika Zangelmi[1], Alessio Peracchi[1], Andrea Secchi[1], Eugenia Polverini[5], Gabriele Giachin[2], Roberto Battistutta[2], Roberto Contestabile[3], Riccardo Percudani[1]

[1]Department of Chemistry, Life Sciences and Environmental Sustainability, University of Parma, Parma, Italy

[2]Department of Chemical Sciences, University of Padua, Padova, Italy

[3]Istituto Pasteur Italia-Fondazione Cenci Bolognetti and Department of Biochemical Sciences "A. Rossi Fanelli", Sapienza University of Rome, Rome, Italy

[4]Institute of Molecular Biology and Pathology, Italian National Research Council, Rome, Italy

[5]Department of Mathematical, Physical and Computer Sciences, University of Parma, Parma, Italy

Supplementary Table 1: PLP-dependent enzyme set used in OSMES[a]

Entry	Gene name	EC number	Organism
**Q3UX83**	Accsl	N/A	Mus musculus
**Q8VCN5**	Cth	4.4.1.1	Mus musculus
**P05201**	Got1	2.6.1.1; 2.6.1.3	Mus musculus
**Q9Z2Y8**	Plpbp	N/A	Mus musculus
**Q9QZX7**	Srr	5.1.1.18; 4.3.1.18; 4.3.1.17	Mus musculus
**Q8BH55**	Thnsl1	N/A	Mus musculus
**Q99K85**	Psat1	2.6.1.52	Mus musculus
**Q9WUB3**	Pygm	2.4.1.1	Mus musculus
**Q14CH1**	Mocos	2.8.1.9	Mus musculus
**Q8CI94**	Pygb	2.4.1.1	Mus musculus
**Q8QZR1**	Tat	2.6.1.5	Mus musculus
**A2AIG8**	Accs	N/A	Mus musculus
**Q8BG54**	Sptlc3	2.3.1.50	Mus musculus
**Q9ET01**	Pygl	2.4.1.1	Mus musculus
**Q9JLI6**	Scly	4.4.1.16	Mus musculus
**Q8R0X7**	Sgpl1	4.1.2.27	Mus musculus
**O35423**	Agxt	2.6.1.51; 2.6.1.44	Mus musculus
**Q6P6M7**	Sepsecs	2.9.1.2	Mus musculus
**P97363**	Sptlc2	2.3.1.50	Mus musculus
**P29758**	Oat	2.6.1.13	Mus musculus
**Q8R238**	Sdsl	4.3.1.17; 4.3.1.19	Mus musculus
**Q8VBT2**	Sds	4.3.1.17; 4.3.1.19	Mus musculus
**Q9CXF0**	Kynu	3.7.1.3	Mus musculus
**Q9Z1J3**	Nfs1	2.8.1.7	Mus musculus
**Q8BGT5**	Gpt2	2.6.1.2	Mus musculus
**Q71RI9**	Kyat3	2.6.1.7; 4.4.1.13; 2.6.1.63	Mus musculus
**P08680**	Alas2	2.3.1.37	Mus musculus
**Q9WVM8**	Aadat	2.6.1.39; 2.6.1.7	Mus musculus
**Q8VC19**	Alas1	2.3.1.37	Mus musculus
**Q8QZR5**	Gpt	2.6.1.2	Mus musculus
**Q91W43**	Gldc	1.4.4.2	Mus musculus
**Q7TSV6**	Got1l1	2.6.1.1	Mus musculus
**Q8BWU8**	Etnppl	4.2.3.2	Mus musculus
**O88986**	Gcat	2.3.1.29	Mus musculus
**Q8BTY1**	Kyat1	2.6.1.7; 4.4.1.13; 2.6.1.64	Mus musculus

**Q9CZN7**	Shmt2	2.1.2.1	Mus musculus
**P61922**	Abat	2.6.1.19; 2.6.1.22	Mus musculus
**Q8R1K4**	Phykpl	4.2.3.134	Mus musculus
**P00860**	Odc1	4.1.1.17	Mus musculus
**Q3UEG6**	Agxt2	2.6.1.44; 2.6.1.40	Mus musculus
**P50431**	Shmt1	2.1.2.1	Mus musculus
**Q80WP8**	Gadl1	4.1.1.11; 4.1.1.29	Mus musculus
**P05202**	Got2	2.6.1.1; 2.6.1.7	Mus musculus
**P48318**	Gad1	4.1.1.15	Mus musculus
**O88533**	Ddc	4.1.1.28	Mus musculus
**Q9DBE0**	Csad	4.1.1.29; 4.1.1.11	Mus musculus
**P24288**	Bcat1	2.6.1.42	Mus musculus
**P48320**	Gad2	4.1.1.15	Mus musculus
**P23738**	Hdc	4.1.1.22	Mus musculus
**O35855**	Bcat2	2.6.1.42	Mus musculus
**Q91WT9**	Cbs	4.2.1.22	Mus musculus
**Q80W22**	Thnsl2	4.2.3.-	Mus musculus
**Q6XPS7**	Tha1	N/A	Mus musculus
**P17174**	GOT1	2.6.1.1; 2.6.1.3	Homo sapiens
**Q4AC99**	ACCSL	N/A	Homo sapiens
**Q96QU6**	ACCS	N/A	Homo sapiens
**O95470**	SGPL1	4.1.2.27	Homo sapiens
**Q96EN8**	MOCOS	2.8.1.9	Homo sapiens
**O94903**	PLPBP	N/A	Homo sapiens
**Q9Y697**	NFS1	2.8.1.7	Homo sapiens
**P06737**	PYGL	2.4.1.1	Homo sapiens
**Q99259**	GAD1	4.1.1.15	Homo sapiens
**Q16773**	KYAT1	2.6.1.7; 4.4.1.13; 2.6.1.64	Homo sapiens
**O75600**	GCAT	2.3.1.29	Homo sapiens
**P00505**	GOT2	2.6.1.1; 2.6.1.7	Homo sapiens
**P13196**	ALAS1	2.3.1.37	Homo sapiens
**Q8N5Z0**	AADAT	2.6.1.39; 2.6.1.7	Homo sapiens
**Q8TD30**	GPT2	2.6.1.2	Homo sapiens
**Q8NHS2**	GOT1L1	2.6.1.1	Homo sapiens
**P11216**	PYGB	2.4.1.1	Homo sapiens
**P11217**	PYGM	2.4.1.1	Homo sapiens
**Q9HD40**	SEPSECS	2.9.1.2	Homo sapiens

**Q9GZT4**	SRR	5.1.1.18; 4.3.1.18; 4.3.1.17	Homo sapiens
**Q9NUV7**	SPTLC3	2.3.1.50	Homo sapiens
**Q9Y617**	PSAT1	2.6.1.52	Homo sapiens
**Q96I15**	SCLY	4.4.1.16	Homo sapiens
**P20132**	SDS	4.3.1.17; 4.3.1.19	Homo sapiens
**P04181**	OAT	2.6.1.13	Homo sapiens
**O15382**	BCAT2	2.6.1.42	Homo sapiens
**P80404**	ABAT	2.6.1.19; 2.6.1.22	Homo sapiens
**Q05329**	GAD2	4.1.1.15	Homo sapiens
**Q6YP21**	KYAT3	2.6.1.7; 4.4.1.13; 2.6.1.63	Homo sapiens
**Q16719**	KYNU	3.7.1.3	Homo sapiens
**P34897**	SHMT2	2.1.2.1	Homo sapiens
**P22557**	ALAS2	2.3.1.37	Homo sapiens
**P24298**	GPT	2.6.1.2	Homo sapiens
**Q6ZQY3**	GADL1	4.1.1.11; 4.1.1.29	Homo sapiens
**P23378**	GLDC	1.4.4.2	Homo sapiens
**Q9BYV1**	AGXT2	2.6.1.44; 2.6.1.40	Homo sapiens
**P34896**	SHMT1	2.1.2.1	Homo sapiens
**P11926**	ODC1	4.1.1.17	Homo sapiens
**Q8IUZ5**	PHYKPL	4.2.3.134	Homo sapiens
**P19113**	HDC	4.1.1.22	Homo sapiens
**Q8TBG4**	ETNPPL	4.2.3.2	Homo sapiens
**P32929**	CTH	4.4.1.1	Homo sapiens
**P35520**	CBS	4.2.1.22	Homo sapiens
**P20711**	DDC	4.1.1.28	Homo sapiens
**P54687**	BCAT1	2.6.1.42	Homo sapiens
**Q9Y600**	CSAD	4.1.1.29; 4.1.1.11	Homo sapiens
**P17735**	TAT	2.6.1.5	Homo sapiens
**Q96GA7**	SDSL	4.3.1.19; 4.3.1.17	Homo sapiens
**P21549**	AGXT	2.6.1.51; 2.6.1.44	Homo sapiens
**O15270**	SPTLC2	2.3.1.50	Homo sapiens
**Q86YJ6**	THNSL2	4.2.3.-	Homo sapiens
**Q8IYQ7**	THNSL1	N/A	Homo sapiens

[a]Source data are provided as a Source Data file.

Supplementary Table 2: Enzyme-substrate combination used for OSMES validation.

code[a]	Gene	Entry	Organism	Reaction	EC number
**CSU**	CSAD	Q9Y600	*Homo sapiens*	Decarboxylase	4.1.1.29; 4.1.1.11
	Csad	Q9DBE0	*Mus musculus*	Decarboxylase	4.1.1.29; 4.1.1.11
	GADL1	Q6ZQY3	*Homo sapiens*	Decarboxylase	4.1.1.11; 4.1.1.29
	Gadl1	Q80WP8	*Mus musculus*	Decarboxylase	4.1.1.11; 4.1.1.29
**CYT**	CTH	P32929	*Homo sapiens*	Other	4.4.1.1
	Cth	Q8VCN5	*Mus musculus*	Other	4.4.1.1
**DMA**	AGXT2	Q9BYV1	*Homo sapiens*	Aminotransferase	2.6.1.44; 2.6.1.40
	Agxt2	Q3UEG6	*Mus musculus*	Aminotransferase	2.6.1.44; 2.6.1.40
**EAP**	ETNPPL	Q8TBG4	*Homo sapiens*	Other	4.2.3.2
	Etnppl	Q8BWU8	*Mus musculus*	Other	4.2.3.2
**GLU**	GAD1	Q99259	*Homo sapiens*	Decarboxylase	4.1.1.15
	GAD2	Q05329	*Homo sapiens*	Decarboxylase	4.1.1.15
	Gad1	P48318	*Mus musculus*	Decarboxylase	4.1.1.15
	Gad2	P48320	*Mus musculus*	Decarboxylase	4.1.1.15
**HIS**	HDC	P19113	*Homo sapiens*	Decarboxylase	4.1.1.22
	Hdc	P23738	*Mus musculus*	Decarboxylase	4.1.1.22
**ILE**	BCAT1	P54687	*Homo sapiens*	Aminotransferase	2.6.1.42
	BCAT2	O15382	*Homo sapiens*	Aminotransferase	2.6.1.42
	Bcat1	P24288	*Mus musculus*	Aminotransferase	2.6.1.42
	Bcat2	O35855	*Mus musculus*	Aminotransferase	2.6.1.42
**KYN**	AADAT	Q8N5Z0	*Homo sapiens*	Aminotransferase	2.6.1.39; 2.6.1.7
	Aadat	Q9WVM8	*Mus musculus*	Aminotransferase	2.6.1.39; 2.6.1.7
	KYAT1	Q16773	*Homo sapiens*	Aminotransferase	2.6.1.7; 4.4.1.13; 2.6.1.64
	KYAT3	Q6YP21	*Homo sapiens*	Aminotransferase	2.6.1.7; 4.4.1.13; 2.6.1.63
	Kyat1	Q8BTY1	*Mus musculus*	Aminotransferase	2.6.1.7; 4.4.1.13; 2.6.1.64
	Kyat3	Q71RI9	*Mus musculus*	Aminotransferase	2.6.1.7; 4.4.1.13; 2.6.1.63
	KYNU	Q16719	*Homo sapiens*	Other	3.7.1.3
	Kynu	Q9CXF0	*Mus musculus*	Other	3.7.1.3
**ORN**	ODC1	P11926	*Homo sapiens*	Decarboxylase	4.1.1.17
	Odc1	P00860	*Mus musculus*	Decarboxylase	4.1.1.17
**PHL**	PHYKPL	Q8IUZ5	*Homo sapiens*	Other	4.2.3.134
	Phykpl	Q8R1K4	*Mus musculus*	Other	4.2.3.134
**SER**	SHMT1	P34896	*Homo sapiens*	Aldolase[b]	2.1.2.1
	SHMT2	P34897	*Homo sapiens*	Aldolase[b]	2.1.2.1
	Shmt1	P50431	*Mus musculus*	Aldolase[b]	2.1.2.1
	Shmt2	Q9CZN7	*Mus musculus*	Aldolase[b]	2.1.2.1
**SPL**	SGPL1	O95470	*Homo sapiens*	Aldolase	4.1.2.27
	Sgpl1	Q8R0X7	*Mus musculus*	Aldolase	4.1.2.27

**TYR**	TAT	P17735	*Homo sapiens*	Aminotransferase	2.6.1.5
	Tat	Q8QZR1	*Mus musculus*	Aminotransferase	2.6.1.5
	DDC	P20711	*Homo sapiens*	Decarboxylase	4.1.1.28
	Ddc	O88533	*Mus musculus*	Decarboxylase	4.1.1.28

[a] three-letter code of the substrate used in the pdbqt files.
[b] SHMT catalyzes the transfer of Cβ of serine to tetrahydrofolate (THF) although it can catalyze an aldolase reaction in the absence of THF.

Supplementary Table 3: Significant coevolutionary association of carnitine biosynthesis enzymes TMLD and BBD with PLP-dependent enzymes according to cotr analysis[a].

**5485575at2759 (TMLD or BBD)**											
OG1	*OG2*	*species*	*t1*	*t2*	*c*	*d*	*k*	*cotr_score*	*P_value*[b]	*P_value(adj)*	
884390at2759	5485575at2759	1929	43	52	12	0	12	0.145	3.22E-10	7.56E-03	ornithine aminotransferase
345661at2759	5485575at2759	1929	67	52	15	2	13	0.123	7.20E-09	1.66E-01	ethanolamine-phosphate phospho-lyase
177349at2759	5485575at2759	1929	30	52	9	1	8	0.108	5.97E-07	1.00E+00	glycine decarboxylase
178754at2759	5485575at2759	1929	86	52	12	0	12	0.095	1.37E-06	1.00E+00	low-specificity L-threonine aldolase 2
5471916at2759	5485575at2759	1929	57	52	10	0	10	0.101	1.37E-06	1.00E+00	kynureninase
5474881at2759	5485575at2759	1929	53	52	9	0	9	0.094	6.62E-06	1.00E+00	Aminotransferase
**TMLD**[c]											
OG1	*OG2*	*species*	*t1*	*t2*	*c*	*d*	*k*	*cotr_score*	*P_value*[b]	*P_value(adj)*	
178754at2759	TMLD	1929	86	123	19	0	19	0.100	7.29E-07	1.00E+00	low-specificity L-threonine aldolase 2
884390at2759	TMLD	1929	43	123	13	0	13	0.085	1.09E-06	1.00E+00	ornithine aminotransferase
3024111at2759	TMLD	1929	37	123	12	1	11	0.0738	9.40E-06	1.00E+00	sphingosine-1-phosphate lyase 1
**BBD**[c]											
OG1	*OG2*	*species*	*t1*	*t2*	*c*	*d*	*k*	*cotr_score*	*P_value*[b]	*P_value(adj)*	
178754at2759	BBD	1929	86	77	16	0	16	109	9.22E-08	1.00E+00	low-specificity L-threonine aldolase 2
884390at2759	BBD	1929	43	77	12	1	11	101	4.10E-07	1.00E+00	ornithine aminotransferase
5487987at2759	BBD	1929	46	77	12	1	11	0.098	8.63E-07	1.00E+00	cystathionine beta-synthase-like
5471916at2759	BBD	1929	57	77	11	0	11	0.089	8.36E-06	1.00E+00	kynureninase
**T_B (TMLD and BBD)**[c]											
OG1	*OG2*	*species*	*t1*	*t2*	*c*	*d*	*k*	*cotr_score*	*P_value*[b]	*P_value(adj)*	
178754at2759	T_B	1929	86	147	23	0	23	110	2.79E-08	6.38E-01	low-specificity L-threonine aldolase 2

[a]Coevolutionary associations were obtained with the cotr program [1] using orthogroups provided by OrthoDB (V. 11) [2].
[b]P-values calculated through the one-tailed Fisher's exact test from the 2 × 2 contingency table of gene presence/absence transitions. P-values(adj) were adjusted for multiple tests using the Holm correction.
[c]The splitting of the 5485575at2759 group into separated TMLD and BBD groups was obtained through Hidden Markov Models (HMMs) of representative sequence alignments of TMLD and BBD. The 3441 sequences of 5485575at2759 were downloaded from OrthoDB and assigned to the group with the highest HMM similarity using hmmscan. The T_B (TMLD and BBD) group was added to the analysis of vectors describing presence/absence data (1/0) by combining with the logical 'and' the TMDB and BBD vectors. Source data are provided as a Source Data file.

Supplementary Table 4: Kinetic parameters of Tha1, SHMT1 and SHMT2 with different β-hydroxylated amino acids.

Enzyme	Substrate	k_{cat} (s^{-1})	K_m (mM)	k_{cat}/K_m (s^{-1} M^{-1})
Tha1	HTML	2.311 ± 0.05	0.169 ± 0.015	13,695.069 ± 1285.936
	L-*allo*-threonine	1.96 ± 0.067	8.236 ± 0.86	238.005 ± 26.154
	L-threonine	0.375 ± 0.041	37.531 ± 10.873	10.005 ± 3.097
SHMT1	HTML	0.123 ± 0.009	3.793 ± 0.606	32.396 ± 5.672
	L-*allo*-threonine	2.365 ± 0.036	0.226 ± 0.017	10,481.813 ± 828.704
	L-threonine	0.154 ± 0.005	36.522 ± 2.484	4.208 ± 0.316
SHMT2	HTML	0.005 ± 0.000	0.803 ± 0.117	6.311 ± 0.951
	L-*allo*-threonine	0.311 ± 0.007	1.345 ± 0.115	231.56 ± 20.564
	L-threonine	0.035 ± 0.001	41.224 ± 3.428	0.846 ± 0.078

Supplementary Table 5: RMSD values (in Å) calculated between Cα-atoms of matched residues.

RMSD (Å)	C2_chainA	C2_chainB
C2_chainA	-	0.262
F222_chainA	0.255	0.280
1M6S_chainA	1.172	1.148
1M6S_chainB	1.059	1.051
1M6S_chainC	1.119	1.110
1M6S_chainD	1.055	1.031

C2 and F222 refer to the Tha1/HTMLA structures in the two space-groups.
1M6S refers to the structure of *Thermotoga maritima* threonine aldolase.

Supplementary Table 6: Parameters relative to the interaction interfaces as determined by PISA.

Protein	Buried Area (Å^2)	ΔG^{int} (kcal/mol)	ΔG^{int} P-value[a]	HB[a]	SB[a]	CSS[a]	$T\Delta S^{diss}$ (kcal/mol)	ΔG^{diss} (kcal/mol)
ABCD ⇒ AB + CD								
C2	14770	-31.5	-	-	-	-	15.8	5.0
F222	14070	-34.7	-	-	-	-	15.7	6.1
1M6S	13915	-67.6	-	-	-	-	15.5	40.9
AB ⇒ A + B or CD ⇒ C+D (main interface)								
C2	3762.9	-15.0	0.141	29	6	1.000	14.2	14.6
F222	3600.0	-16.8	0.085	28	6	1.000	14.2	15.9
1M6S	3680.0	-14.5	0.253	23	8	1.000	14.0	11.7
AC ⇒ A + C or BD ⇒ B + DC (secondary interface)								
C2	2132.0	-5.3	0.409	6	6	0.225	nd	nd
F222	1986.0	-0.9	0.679	10	4	0.144	nd	nd
1M6S	1910.0	-18.0	0.017	7	0	1.000	13.7	7.4

[a] values determined only for single interfaces.
nd, “not determined” by PISA because of unstable interactions.
C2 and F222 refer to the Tha1/HTMLA structures in the two space-groups. 1M6S refers to the structure of *Thermotoga maritima* threonine aldolase.
HB, number of hydrogen bonds; SB, number of salt bridges.
See main text for the significance of the other parameters.

Supplementary Table 7: CC-CFC with different structure models of Tha1.

rank	Gene	structure	CC-CFC	$\|\sin(\chi_2)\|$	LCC	d	E (kcal/mol)
**2**	Tha1	F222	91	0.754	143	4.55	-8.8
**4**	Tha1	C2	75	0.681	158	4.26	-9.7
**5**	Tha1	AF_only_F222[a]	70	0.754	130	4.48	-9.3
**6**	Tha1	AF_F222[b]	63	0.734	122	4.46	-9.1
**7**	Tha1	AF_aligned_F222[c]	60	0.659	157	4.27	-9.7
**9**	Tha1	AF	51	0.724	111	4.83	-9.1

[a] AlphaFold model obtained with F222 as a unique template.
[b] AlphaFold model obtained adding F222 to template list.
[c] AlphaFold model from AFDB aligned to F222 quaternary structure.
Source data are provided as a Source Data file.

Supplementary Table 8: Experimental details on the SAXS experiment and analysis.

(A) Sample details

Sample name	Threonine aldolase 1 (Tha 1)
Organism	*Mus musculus* (Mouse)
UniProt sequence ID	Q6XPS7
Calculated molecular weight (Da)	41497.28 (monomer), 165989.12 (tetramer)
Total frames (frames used)	26
Protein concentration (mg/mL)	5.5 mg/mL
SEC-SAXS column	Superdex 200 10/300 GL Cytiva
Injection volume, flow rate	250 µL, 0.5 mL/min
Protetin buffer	20 mM Tris, 150 mM NaCl, 1 mM EDTA, 1 mM DTT, pH 7.8

(B) SAXS data collection parameters

Instrument	ESRF BM29
Wavelength (Å)	0.99
q-range (Å)	0.004-0.5
Sample-to-detector distance (m)	2.867
Exposure time	0.5 sec/frame
Temperature (°C)	20
Detector	Pilatus3 X 2M (Dectris)
Flux (photons/s)	2×10^{12}
Beam size (µm)	100 x 100
Sample configuration	1.8 mm quartz glass capillary
Absolute scaling method	Comparison to water in sample capillary
Normalization	To transmitted intensity by beam-stop counter

(C) Structural parameters

Guinier analysis	
I(0)	21.06
R_g (nm)	3.69 ± 0.03
q-range (nm^{-1}), point range	0.0267-0.1230, 14-50
P(r) analysis	
I(0)	20.88
R_g (nm)	3.62
D_{max} ($R_{max,}$ nm)	10.6
q-range (nm^{-1}), point range	0.0177-3.313, 6-592
Porod volume ($Å^3$)	250961
χ^2 [total estimate from GNOM]	0.9102
Mass estimate based on volume (kDa), ratio to predicted tetramer	167.3, 1

(D) Software employed for SAXS data reduction, analysis and interpretation

SAXS data collection and processing	pyFAI, BsxCuBE and Primus (ATSAS 3.2.1)
Shape/bead modelling	DAMMIF (ATSAS 3.2.1)
Atomic structure modelling	CRYSOL (ATSAS 3.2.1)

3D graphic representation	UCSF Chimera 1.15

(E) Shape model-fitting results

DAMMIF (default parameters)	
q range for fitting (nm^{-1})	0.0177-3.313
Symmetry assumption	P2

(F) Atomistic modelling

CRYSOL (default parameters)	
Starting crystal structure	Tha1 tetramer (this study)
χ^2 of the fit	1.145

(G) Small Angle Scattering Biological Data Bank (SASBDB)

SASBDB ID*	SASDSU8

Supplementary Table 9: X-ray diffraction data processing and model refinement statistics.

**Data collection**		
X-ray source	ESRF ID23-2	
Wavelength (Å)	0.8731	
Space group	F222	C2
Cell dimensions		
a, b, c (Å)	83.69, 100.52, 171.26	83.97, 101.64, 95.96
α, β, γ (°)	90.00, 90.00, 90.00	90.00, 116.14, 90.00
Resolution range (Å)	25.71 – 2.26 (2.33 – 2.26)	43.07 – 2.60 (2.72 – 2.60)
R_{merge}	0.084 (0.842)	0.104 (0.731)
R_{meas}	0.088 (0.887)	0.115 (0.804)
R_{pim}	0.027 (0.272)	0.047 (0.325)
Total number of observations	168470 (15409)	109798 (14100)
Total number unique	17051 (1561)	21160 (2662)
Mean(I)/s(I)	16.6 (1.5)	8.9 (1.4)
$CC_{1/2}$	0.998 (0.881)	0.996 (0.786)
Completeness (%)	99.7 (99.9)	94.8 (98.0)
Multiplicity	9.9 (9.9)	5.2 (5.3)
Wilson B estimate (Å^2)	46.4	65.3
**Refinement**		
Resolution range (Å)	25.71 – 2.26	43.07 – 2.60
R_{work}/R_{free} (%)	21.0/24.0	23.4/25.9
Number of atoms		
Protein	2806	5536
Water	57	29
Average B, all atoms (Å^2)	67.0	83.0
r.m.s.d.		
Bond lengths (Å)	0.003	0.002
Bond angles (°)	0.596	0.560
Ramachandran statistics		
Favored (%)	95.4	95.8
Allowed (%)	4.1	3.9
Outliers (%)	0.5	0.3
PDB entry	8PUS	8PUM

Values in parentheses are for the highest-resolution shell.

Supplementary Table 10: Enzyme-substrate combination used for OSMES validation for aldehyde dehydrogenases.

Gene	Entry	Organism	Substrate	code[a]
ALDH18A1	P54886	*Homo sapiens*	glutamate-5-semialdehyde	GSA
ALDH1A1	P00352	*Homo sapiens*		
ALDH1A2	O94788	*Homo sapiens*	retinaldehyde	RET
ALDH1A3	P47895	*Homo sapiens*	retinaldehyde	RET
ALDH1B1	P30837	*Homo sapiens*		
ALDH1L1	O75891	*Homo sapiens*		
ALDH1L2	Q3SY69	*Homo sapiens*		
ALDH2	P05091	*Homo sapiens*		
ALDH3A1	P30838	*Homo sapiens*		
ALDH3A2	P51648	*Homo sapiens*		
ALDH3B1	P43353	*Homo sapiens*		
ALDH3B2	P48448	*Homo sapiens*		
ALDH4A1	P30038	*Homo sapiens*	glutamate-5-semialdehyde	GSA
ALDH5A1	P51649	*Homo sapiens*	succinic semialdehyde	SSA
ALDH6A1	Q02252	*Homo sapiens*		
ALDH7A1	P49419	*Homo sapiens*		
ALDH8A1	Q9H2A2	*Homo sapiens*	2-aminomuconic semialdehyde	ACM
ALDH9A1	P49189	*Homo sapiens*	4-trimethylaminobutyraldehyde	TMABA
GAPDH	P04406	*Homo sapiens*	glyceraldehyde 3-phosphate	GAP
GAPDHS	O14556	*Homo sapiens*		
Aldh18a1	Q9Z110	*Mus musculus*	glutamate-5-semialdehyde	GSA
Aldh1a1	P24549	*Mus musculus*		
Aldh1a2	Q62148	*Mus musculus*	retinaldehyde	RET
Aldh1a3	Q9JHW9	*Mus musculus*	retinaldehyde	RET
Aldh1a7	O35945	*Mus musculus*		
Aldh1b1	Q9CZS1	*Mus musculus*		
Aldh1l1	Q8R0Y6	*Mus musculus*		
Aldh1l2	Q8K009	*Mus musculus*		
Aldh2	P47738	*Mus musculus*		
Aldh3a1	P47739	*Mus musculus*		
Aldh3a2	P47740	*Mus musculus*		
Aldh3b1	Q80VQ0	*Mus musculus*		

Aldh3b2	E9Q3E1	*Mus musculus*		
Aldh3b3	J3QMK6	*Mus musculus*		
Aldh4a1	Q8CHT0	*Mus musculus*	glutamate-5-semialdehyde	GSA
Aldh5a1	Q8BWF0	*Mus musculus*	succinic semialdehyde	SSA
Aldh6a1	Q9EQ20	*Mus musculus*		
Aldh7a1	Q9DBF1	*Mus musculus*		
Aldh8a1	Q8BH00	*Mus musculus*	2-aminomuconic semialdehyde	ACM
Aldh9a1	Q9JLJ2	*Mus musculus*	4-trimethylaminobutyraldehyde	TMABA
Gapdh	P16858	*Mus musculus*	glyceraldehyde 3-phosphate	GAP
Gapdhs	Q64467	*Mus musculus*		

[a] code of the substrate used in the pdbqt files. Source data are provided as a Source Data file.

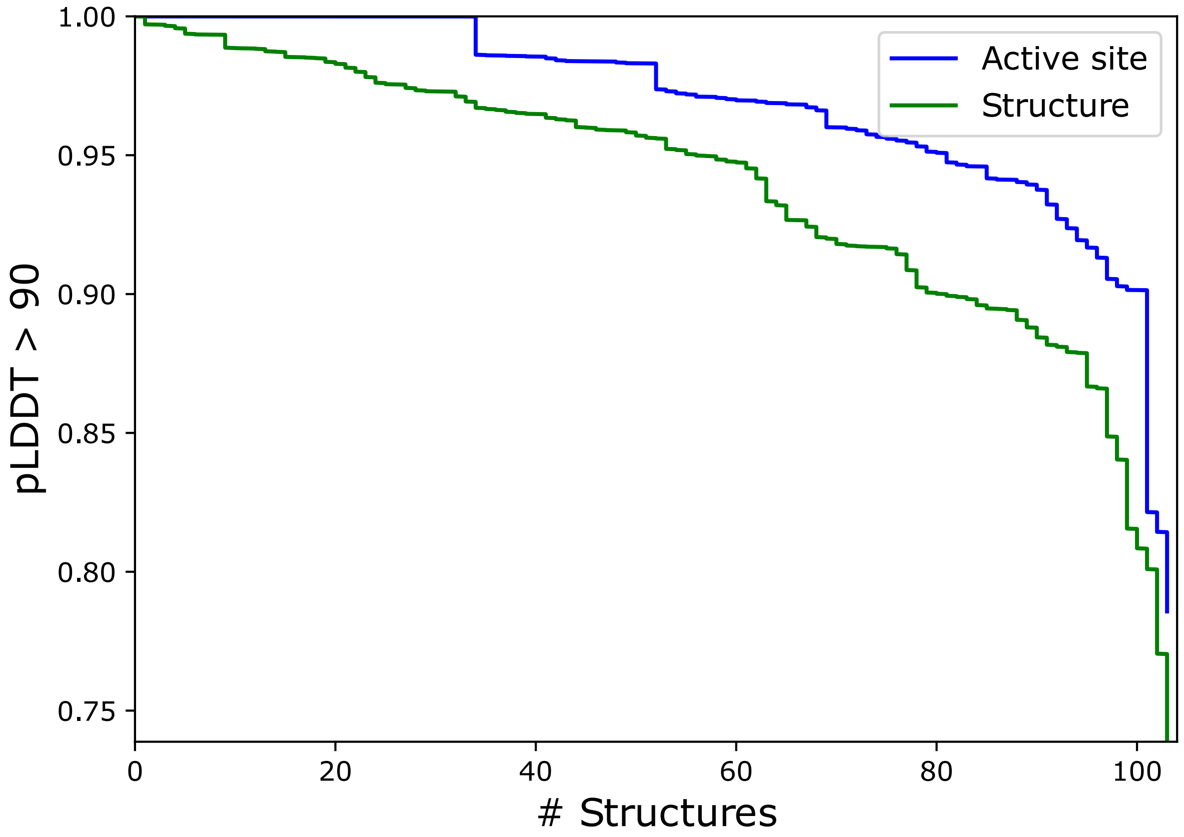

Supplementary Figure 1: Cumulative frequency curve of pLDDT values in the AlphaFold models for human and mouse PLPomes.

Cumulative frequency curves of the residues with very high confidence (pLDDT > 90) of the whole structure (green) or near the active site (<5Å from catalytic lysine; blue) of the entire enzyme set (103 structures).

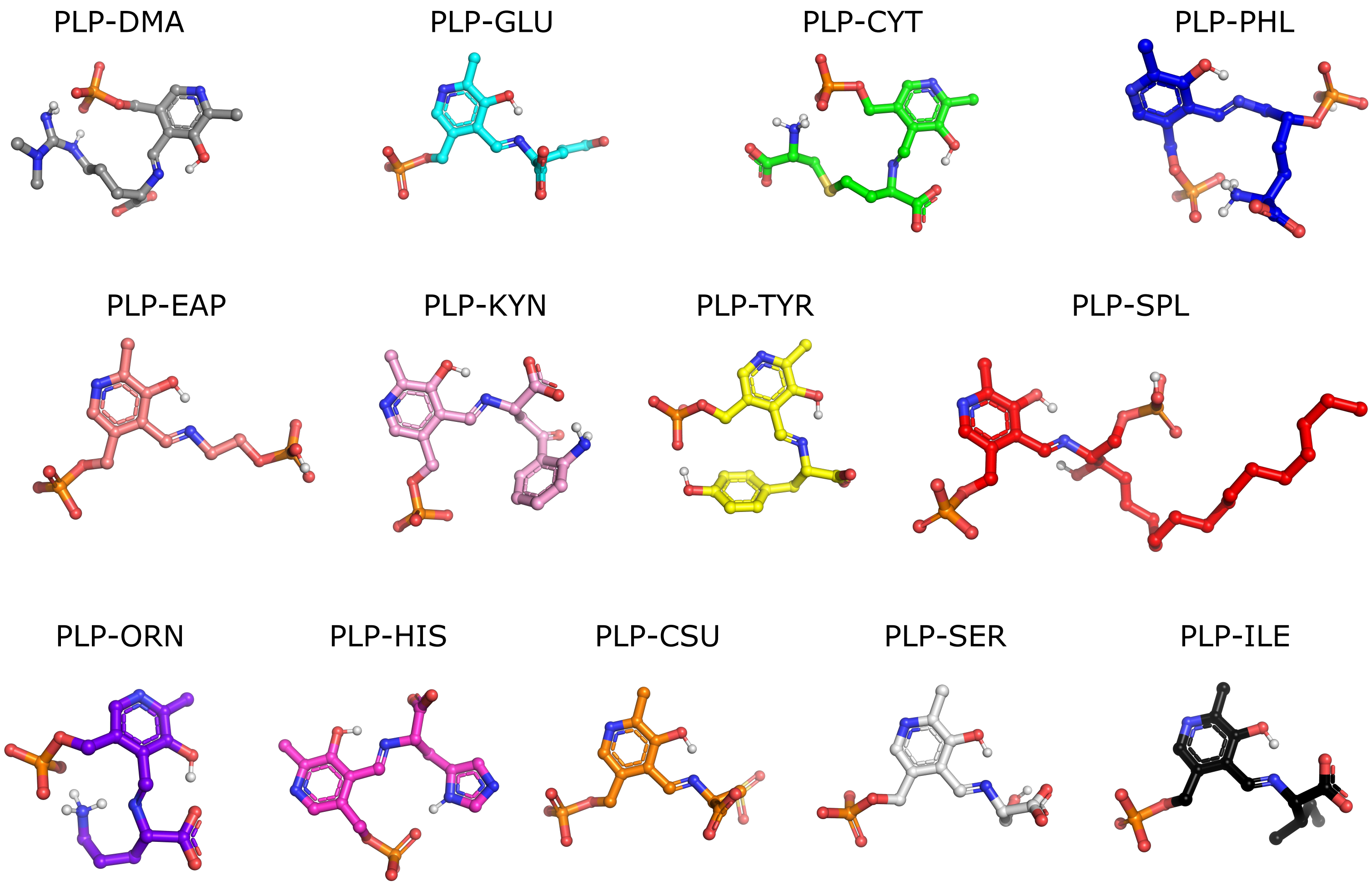

Supplementary Figure 2: Validation library of substrate-PLP complexes.

Substrate-PLP complexes (external aldimine) considered for the selection of the best classification method. The energy-minimized conformation of each substrate is shown in ball-and-stick representation. Carbon atoms are colored according to **Fig. 2e**; non-carbon atoms are colored according to CPK. Shown are PLP-bound asymmetric-L-dimethylarginine (PLP-DMA), L-glutamate (PLP-GLU), L-cystathionine (PLP-CYT), 5-phosphohydroxy-L-Lysine (PLP-PHL), phosphoethanolamine (PLP-EAP), L-kynurenine (PLP-KYN), L-tyrosine (PLP-TYR), sphingosine-1-phosphate (PLP-SPL), L-ornithine (PLP-ORN), L-histidine (PLP-HIS), L-cysteinesulfinate (PLP-CSU), L-serine (PLP-SER), L-isoleucine (PLP-ILE). Source data are provided as a Source Data file.

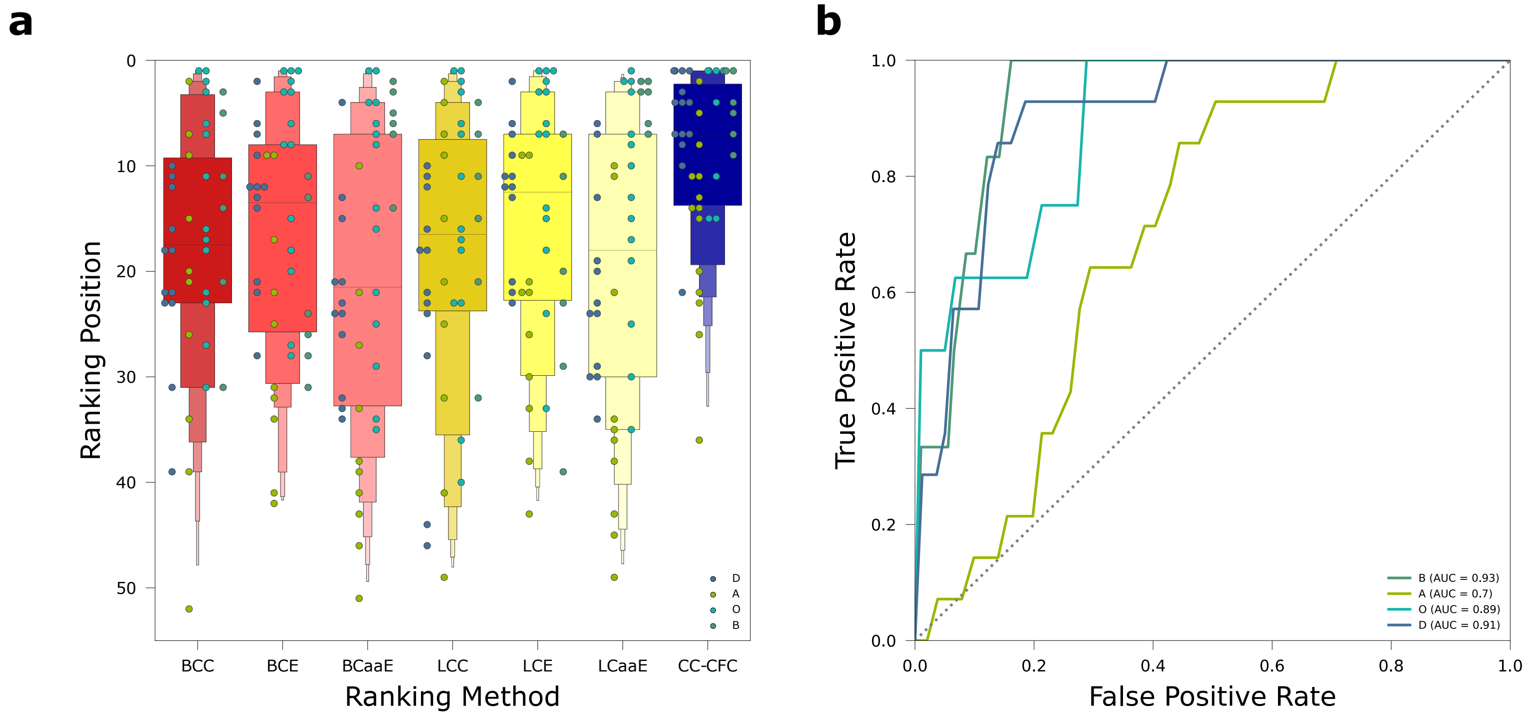

Supplementary Figure 3: OSMES performance with positive controls grouped by reaction type.

a, Letter-value plot showing the distribution of the validation set (n=42) colored according to the 7 ranking methods. BC related methods are colored in red tones; LC related methods are colored in yellow tones; CC-CFC is colored in blue. The band indicates the median, the main box indicates the first and third quartiles with every further minor box splitting the remaining data into two halves. Individual dots representing ranking positions of positive controls (i.e. enzymes known to act on the substrate) are colored according to reaction type (D: decarboxylase; A: aminotransferase; O: other; B: aldolase, including SHMTs). **b**, Receiver operating characteristic curve (ROC) of the CC-CFC method divided according to reaction type. The dotted diagonal represents an area under curve (AUROC) value of 0.5.

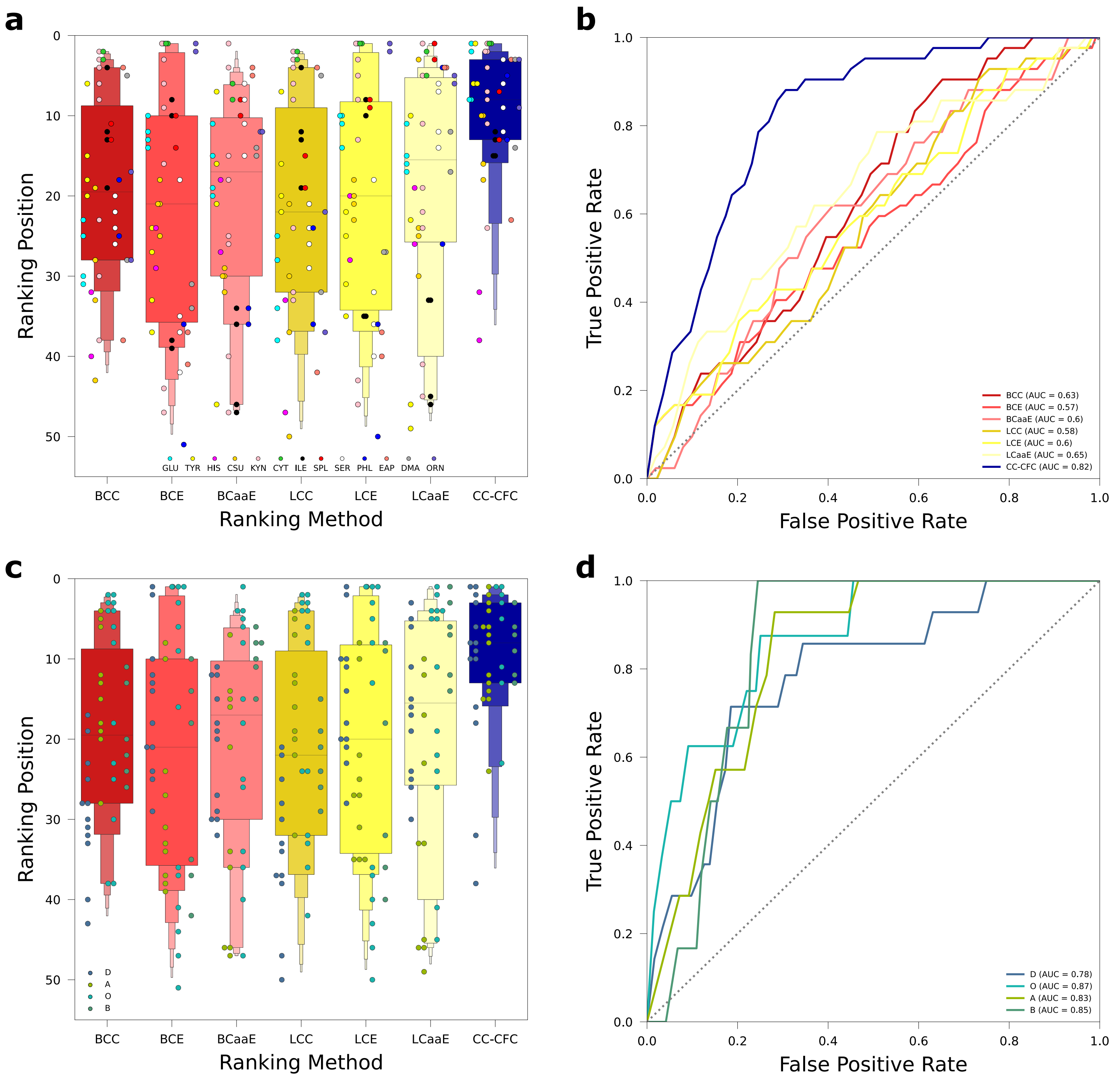

Supplementary Figure 4: OSMES performance with positive controls using AlphaFold Multimer. **a**, **c** Letter-value plot showing the distribution of the validation set (n=42) colored according to the 7 ranking methods with AlphaFold Multimer. The band indicates the median, the main box indicates the first and third quartiles with every further minor box splitting the remaining data into two halves. Individual dots are colored according to substrates (**a**) or according to reaction type (**c**). **b**, Receiver operating characteristic curve (ROC) for the different ranking methods colored as in panel a; **d**, Receiver operating characteristic curve (ROC) of the CC-CFC method colored as in panel c. Source data are provided as a Source Data file.

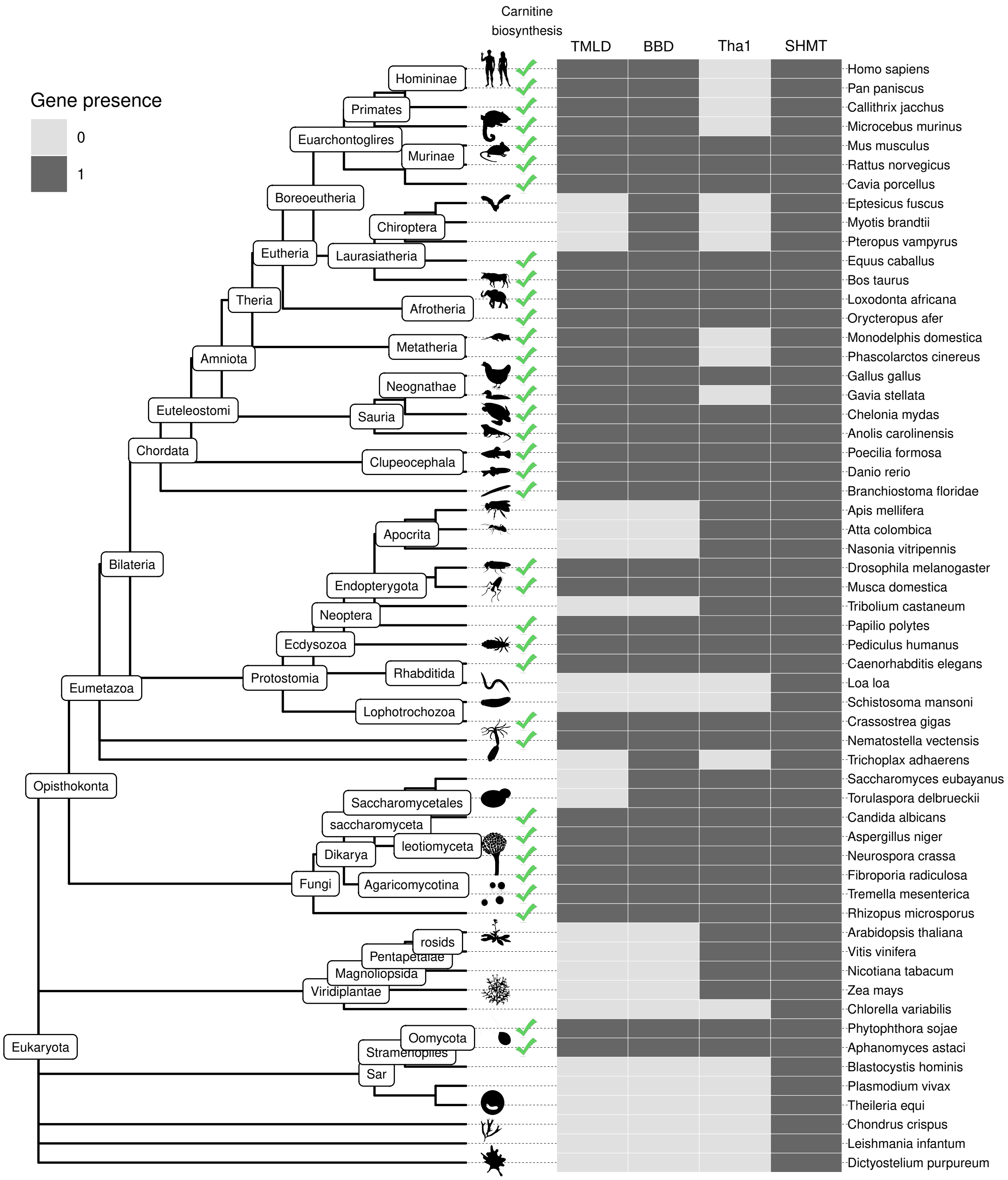

Supplementary Figure 5: Distribution of genes of the carnitine pathway across eukaryote phylogeny.

Presence of the carnitine biosynthetic pathway in selected eukaryotes as deduced from the co-presence of trimethyllysine dioxygenase (TMLD) and ɣ-butyrobetaine dioxygenase (BBD). The distribution of Threonine aldolase (Tha1) and serine hydroxymethyl transferase characterized in this work for hydroxy trimethyl-lysine aldolase (HTMLA) activity are shown for comparison. The presence of genes was assessed by hmmsearch using Hidden Markov Models built on protein alignments (TMLD, BBD) or extracted from B6DB (Tha1, SHMT). The presence of the carnitine biosynthesis pathway is indicated by a green check mark as deduced by the co-presence of TMLD and BBD. The tree represents a simplified scheme of eukaryote phylogeny according to NCBI Taxonomy. PhyloPic silhouettes (https://www.phylopic.org) are added to species names to aid identification. Source data are provided as a Source Data file.

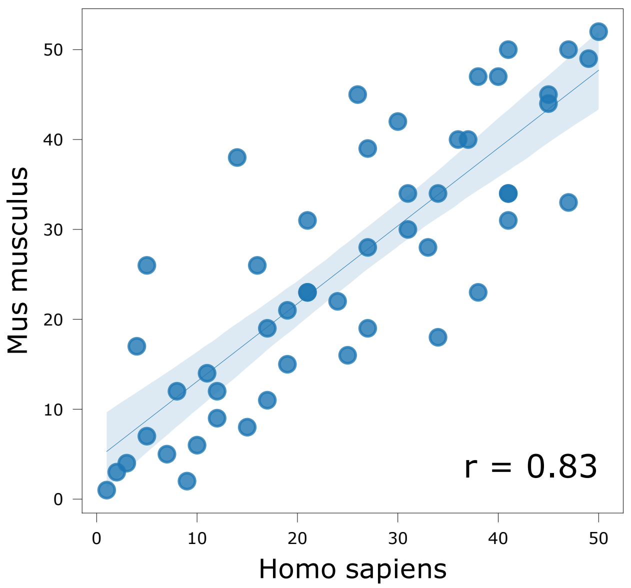

Supplementary Figure 6: Correlation between the ranking of human and mouse orthologous genes.

Scatter plot of the ranking position of each orthologous pair in human (x-axis) and mouse (y axis) CC-CFC ranking. The blue line represents the linear regression of the points; *r* represents the Spearman's rank correlation coefficient.

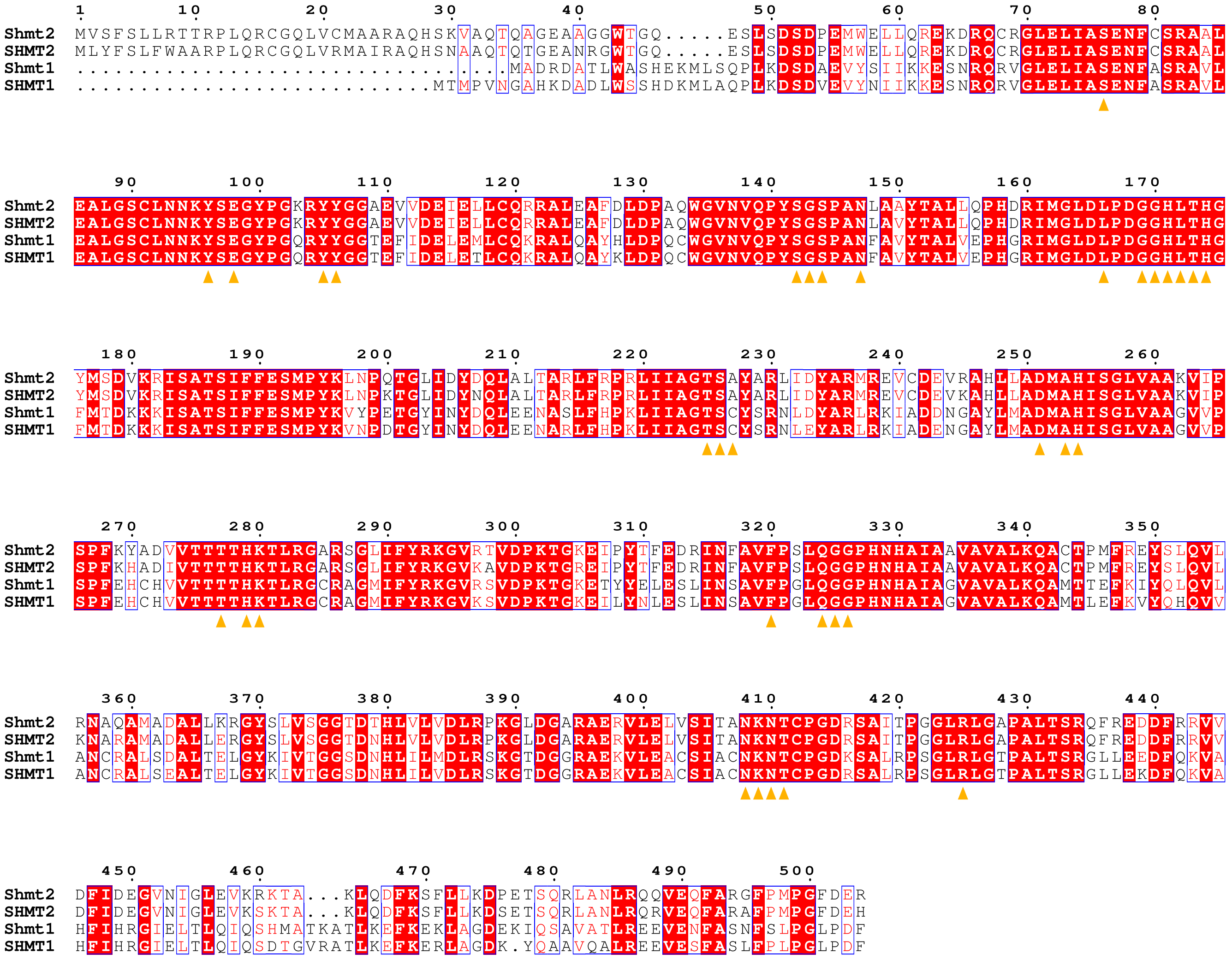

Supplementary Figure 7: Multiple sequence alignment of human and mouse SHMTs.

Multiple sequence alignment of the main isoforms of human and mouse SHMTs obtained with ClustalΩ and visualized with ESPript. Red shading is according to residue conservation, orange triangles indicate residues at ≤ 3.5 Å from the external aldimine (PDB ID: 6FL5). Source data are provided as a Source Data file.

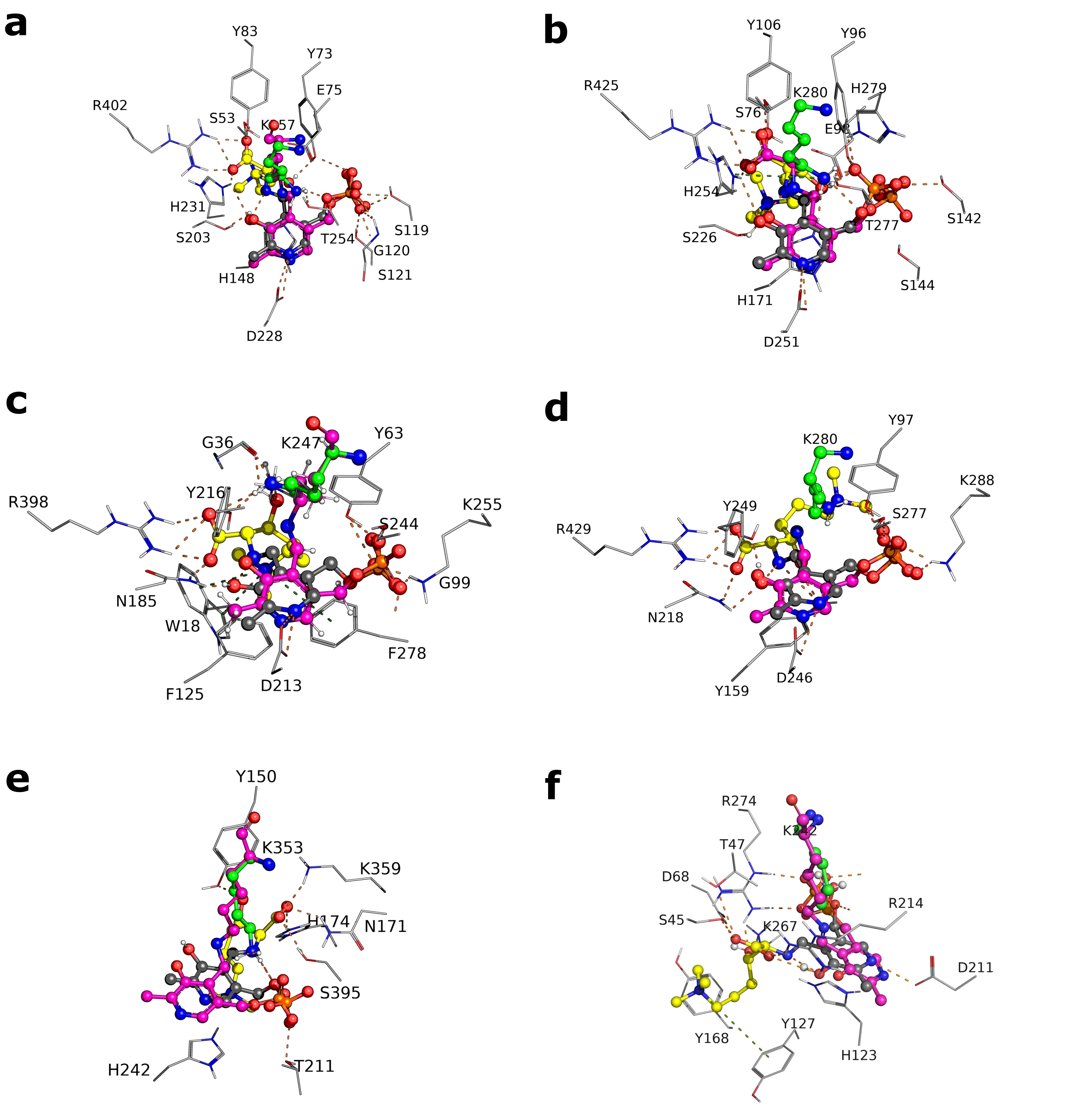

Supplementary Figure 8: Comparison of PLP cofactor positions in docked poses and in experimental structures.

Structural representation of the lowest-energy binding modes among the catalytic clusters obtained by docking of PLP-HTML substrate for SHMT1 (**a**), SHMT2 (**b**), KYAT1 (**c**), KYAT3 (**d**), SGPL1 (**e**), Tha1 (**f**) aligned with experimental structures (PDB IDs: 6FL5, 8AQL, 4WLH, 5VEQ, 4Q6R, 1JG8, respectively) containing PLP as external or internal aldimine (purple). Non-carbon atoms are colored according to CPK convention. The conformations are shown with ball-and-sticks composed by PLP cofactor (gray) covalently bound to HTML (yellow), and flexible catalytic lysine (green). The binding site residues (≤ 3.5 Å from PLP-HTML) are shown in lines labeled with one-letter code and number. Polar interactions between substrate and protein are indicated with yellow dashes, while cation-π interactions are indicated with olive dashes.

The RMSD obtained by the comparison of the PLP atoms are the following: SHMT2: 0.90Å, Tha1: 1.39Å, SHMT1: 0.74Å, SGPL1: 2.09Å, KYAT3: 1.09Å KYAT1: 1.64Å. Source data are provided as a Source Data file.

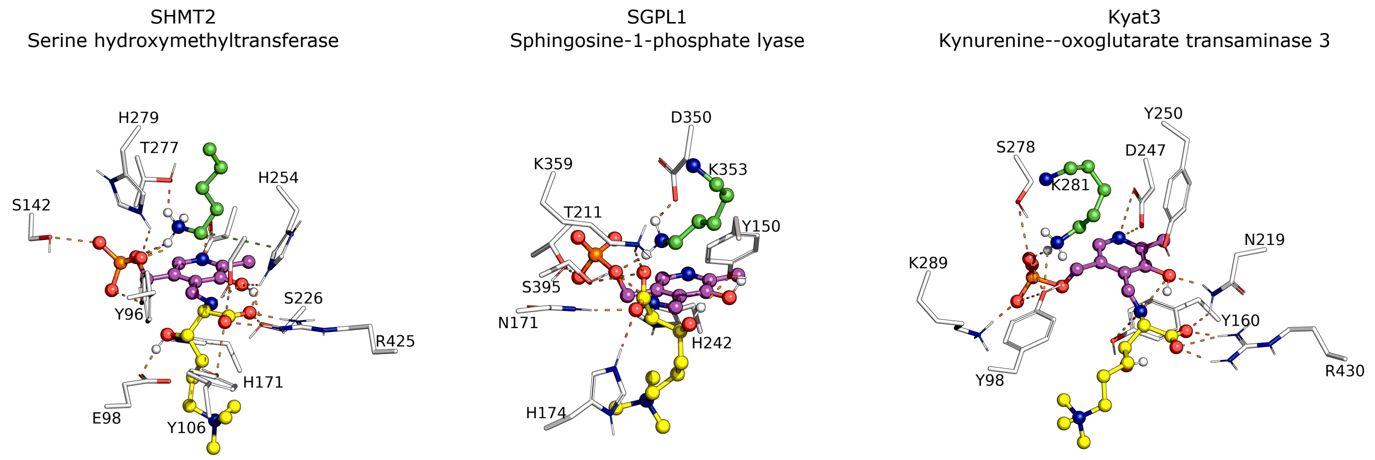

Supplementary Figure 9: Docking poses of SHMT2, SGPL1 and Kyat3 after HTML-OSMES.

Structural representation of the lowest-energy binding modes among the catalytic clusters obtained by docking of HTML-PLP substrate for SHMT2, SGPL1 and Kyat3. Non-carbon atoms are colored according to CPK. The conformations are shown with ball-and-sticks composed by PLP cofactor (gray) covalently bound to HTML (yellow), and flexible catalytic lysine (green). The binding site residues (≤ 3.5 Å from HTML-PLP) are shown in lines labeled with one-letter code and number. Polar interactions between substrate and protein are indicated with yellow dashes, while cation-π interactions are indicated with olive dashes. Source data are provided as a Source Data file.

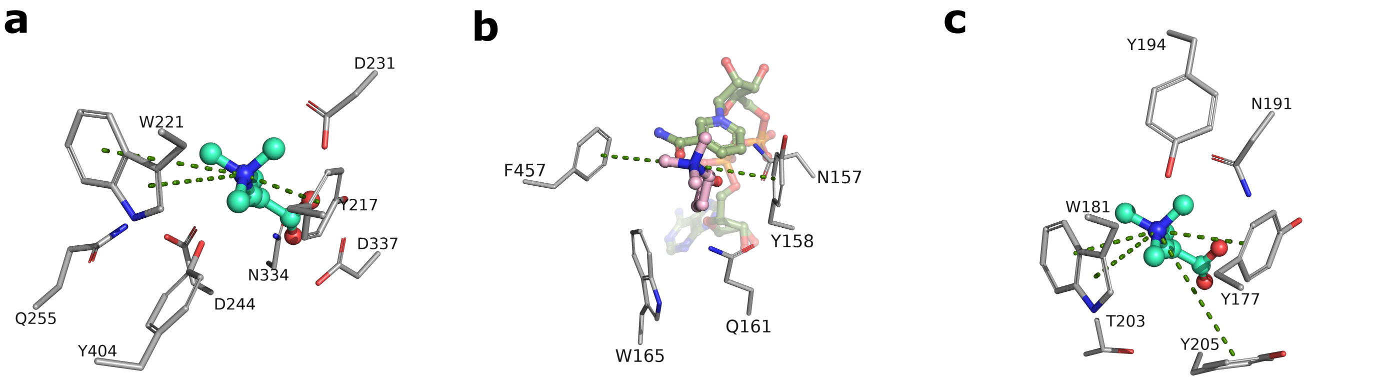

Supplementary Figure 10: Aromatic cage of enzymes involved in carnitine biosynthesis.

a, Structural representation of aromatic cage in the crystal structure of human BBD crystal structure (PDB ID: 3O2G) in complex with the substrate butyrobetaine. **b**, Structural representation of aromatic cage in the AlphaFold model of human TMLD in complex with butyrobetaine from the structural alignment with BBD structure. **c**, Structural representation of aromatic cage in the crystal structure of human TMABADH crystal structure (PDB ID: 6V6R) in complex with the docked substrate TMABA using ADFR. In all panels, cation-π interactions are indicated with olive dashes. Source data are provided as a Source Data file.

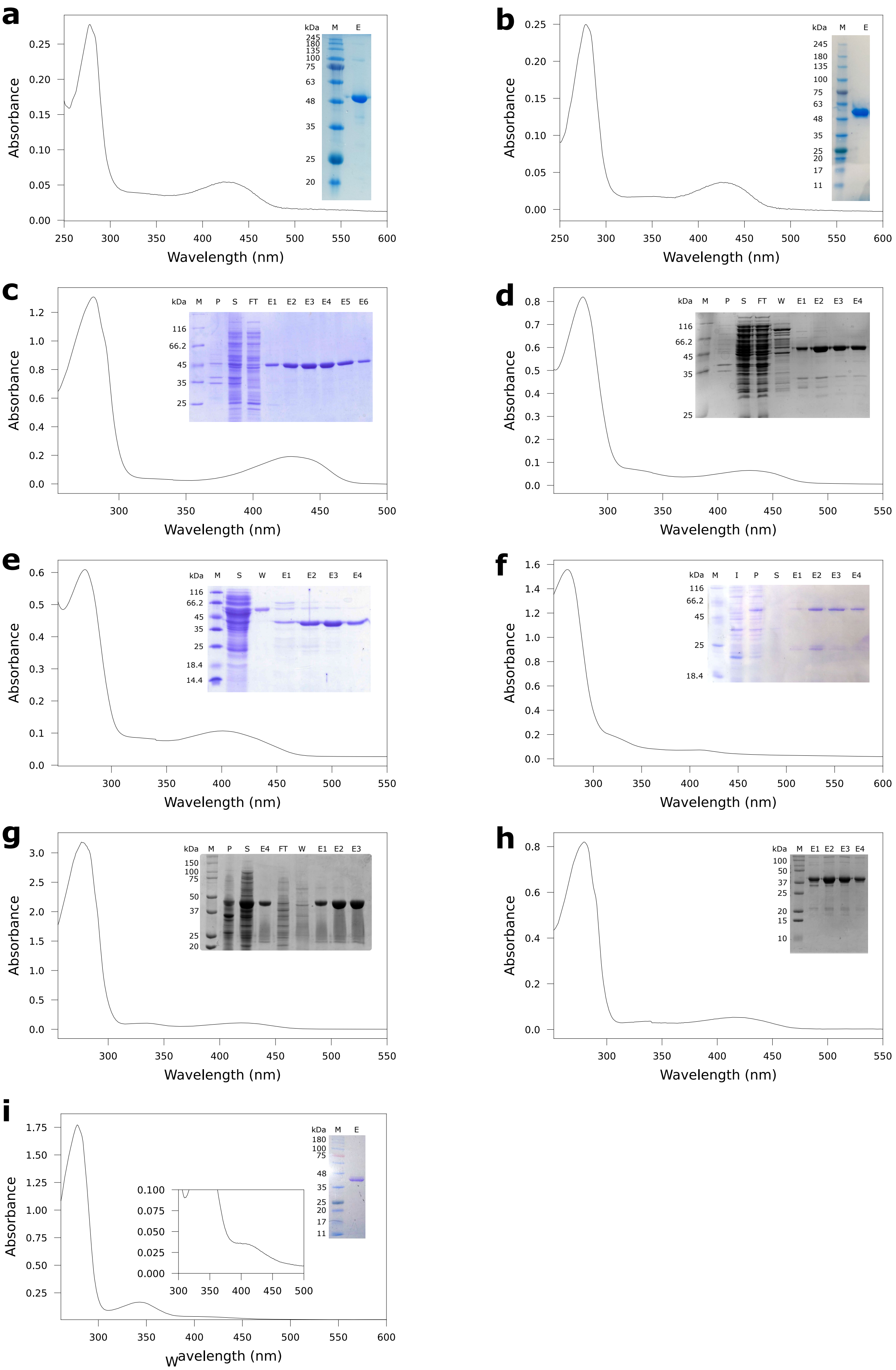

Supplementary Figure 11: SDS-PAGE and UV-Vis spectra of purified enzymes.

UV -Vis spectra of SHMT1 (**a**), SHMT2 (**b**), KYAT1 (**c**), Kyat3 (**d**), Tha1 (**e**), SGPL1 (**f**), Oat (**g**), Thnsl2 (**h**), PSAT1 (**i**), showing a protein peak around 280 nm and a PLP signal around 400-430 nm, characteristic of the ketoenamine tautomer. The ketoenamine peak of PSAT1[3] can be observed with a zoomed view as shown in the inset. Insets show SDS-PAGE of the purification steps (M: marker; I: induced cells; S: soluble fraction; P: pellet; FT: flow-through; W: washing; E: elution). Source data are provided as a Source Data file.

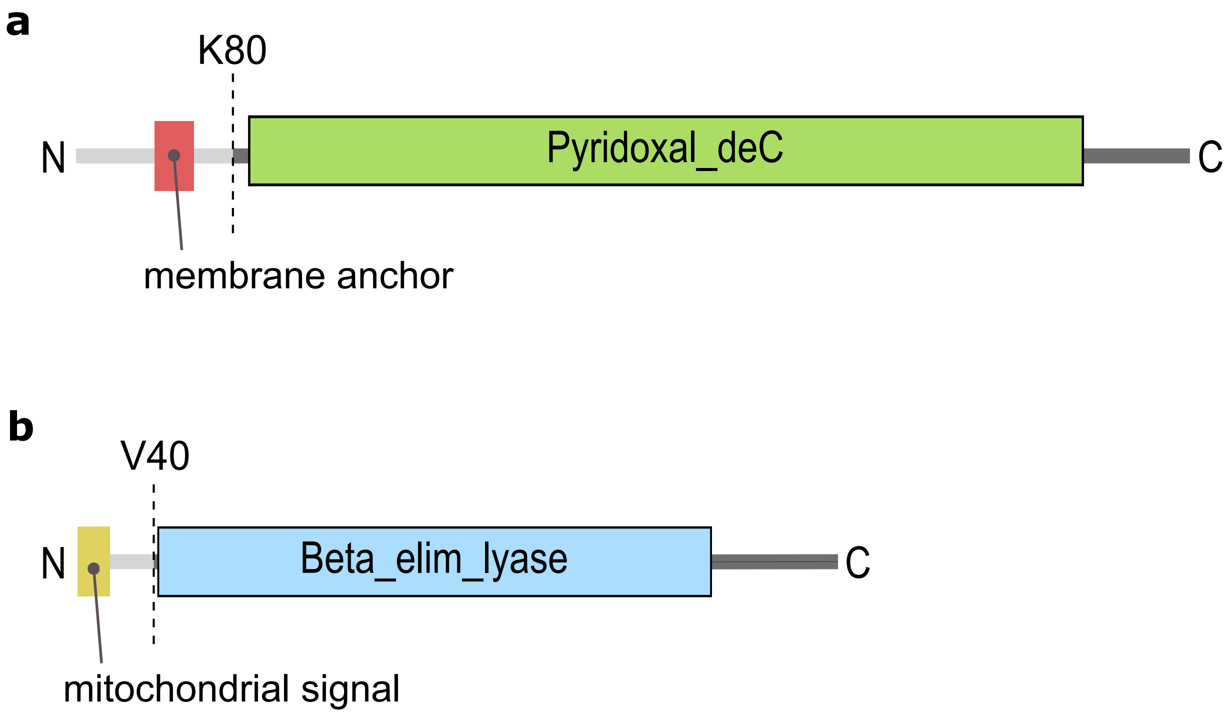

Supplementary Figure 12: Tha1 and SGPL1 N-terminal truncation

a, SGPL1 domain composition according to PFAM. The dashed line indicates membrane anchor truncation for recombinant protein expression. **b**, Tha1 domain composition according to PFAM. The dashed line indicates mitochondrial signal truncation for recombinant protein expression.

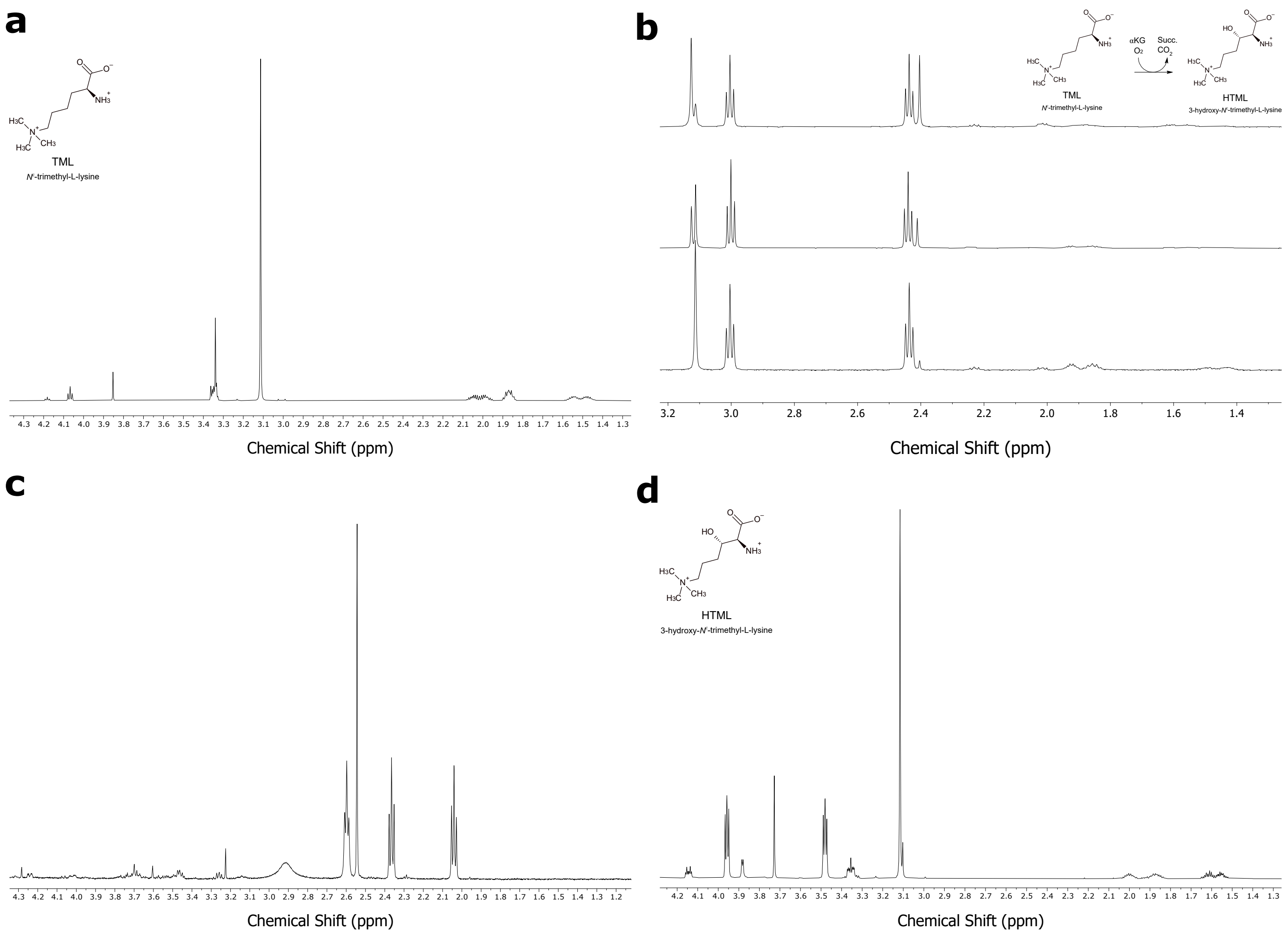

Supplementary Figure 13: Chemo-enzymatic synthesis of HTML

a, ^{1}H NMR spectrum of chemically synthesized trimethyllysine (TML). **b**, ^{1}H NMR spectra of TML after the addition of TMLD enzyme for the enzymatic synthesis of (S,S) HTML. **c**, ^{1}H NMR spectrum of flow-through after HTML purification from reaction mixture. **d**, ^{1}H NMR spectrum of purified HTML used in the activity assays. Source data are provided as a Source Data file.

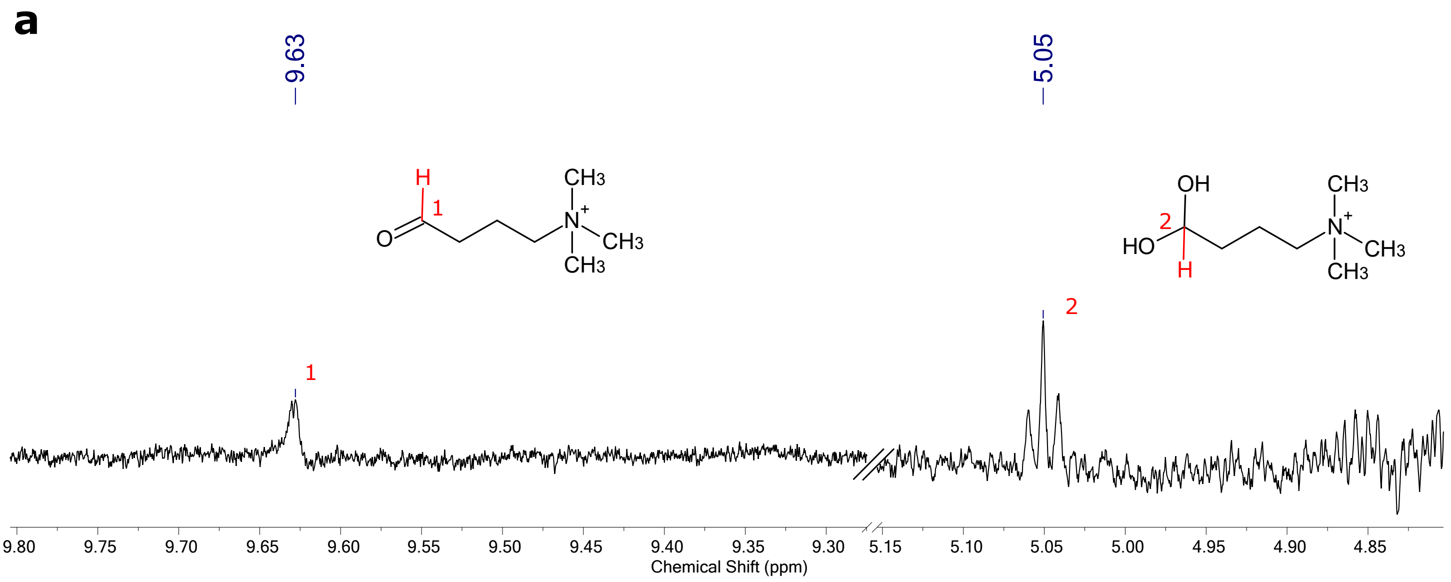

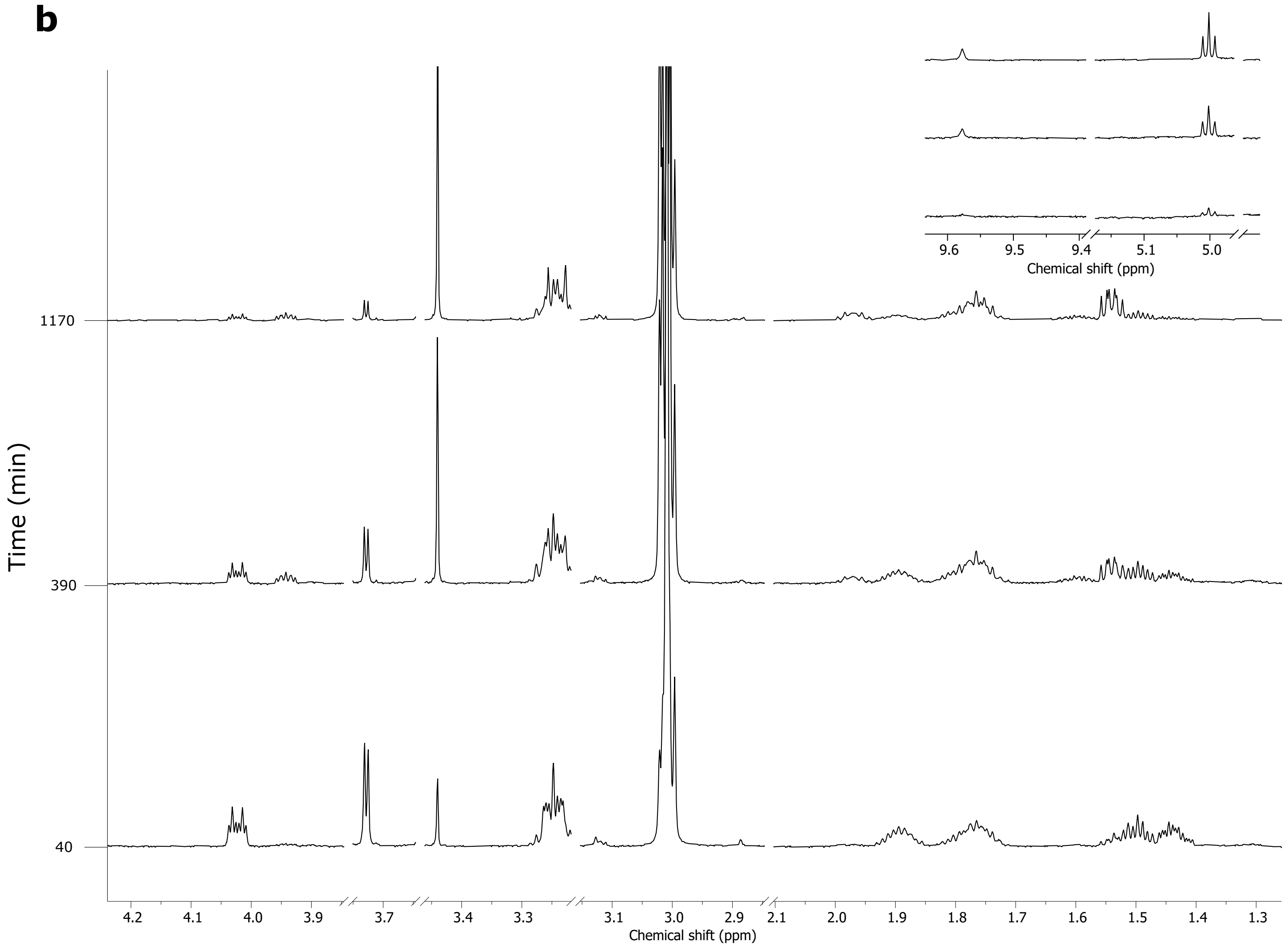

Supplementary Figure 14: ^{1}H NMR spectra of HTMLA reaction

a, Detail of ^{1}H NMR spectrum of aldehyde proton of TMABA after 1100 minutes of SHMT1 reaction in dehydrated (9.63) and hydrated (5.05 ppm) form. **b**, Time-resolved ^{1}H NMR spectra of Tha1 activity in the presence of 5 mM HTML at 40, 390 and 1170 minutes. Inset shows aldehyde proton of TMABA in dehydrated (9.63) and hydrated (5.05 ppm) form. Source data are provided as a Source Data file.

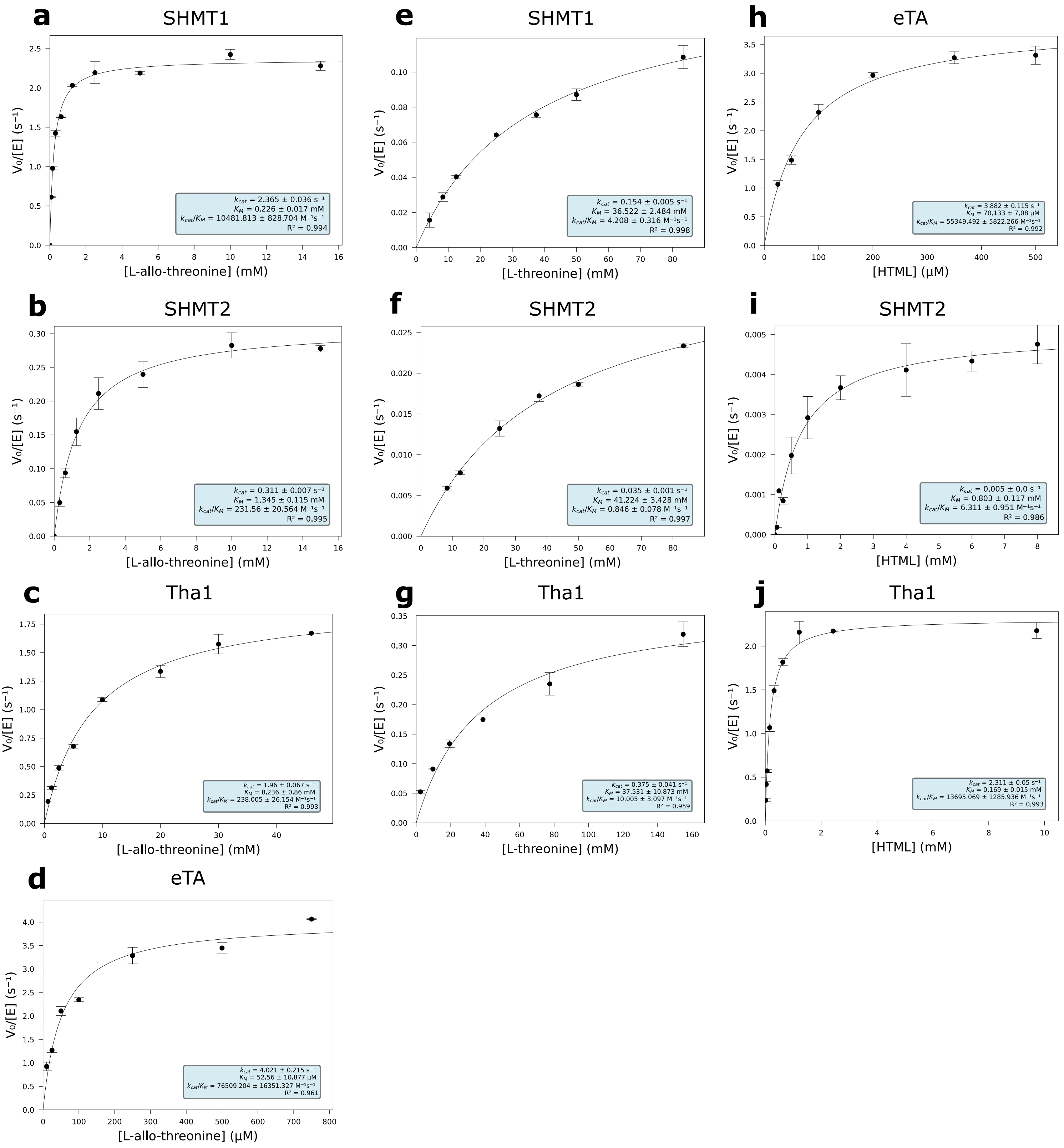

Supplementary Figure 15: Michaelis-Menten fitting of aldol cleavage of human SHMTs, Tha1 and *e*TA. Michaelis -Menten plots of aldolase activity towards: L-*allo*-threonine for SHMT1 (**a**), SHMT2 (**b**), Tha1 (**c**), *e*TA (**d**); L-threonine for SHMT1 (**e**), SHMT2 (**f**), Tha1 (**g**); HTML for *e*TA (**h**), SHMT2 (**i**) and Tha1 (**j**). Data are presented as mean values ± SEM; n=4 independent experiments for each point for panels **a**, **b**, **e**, **f**, **i**; n=3 independent experiments for each point for panels **c**, **d**, **g**, **h**, **j**. Source data are provided as a Source Data file.

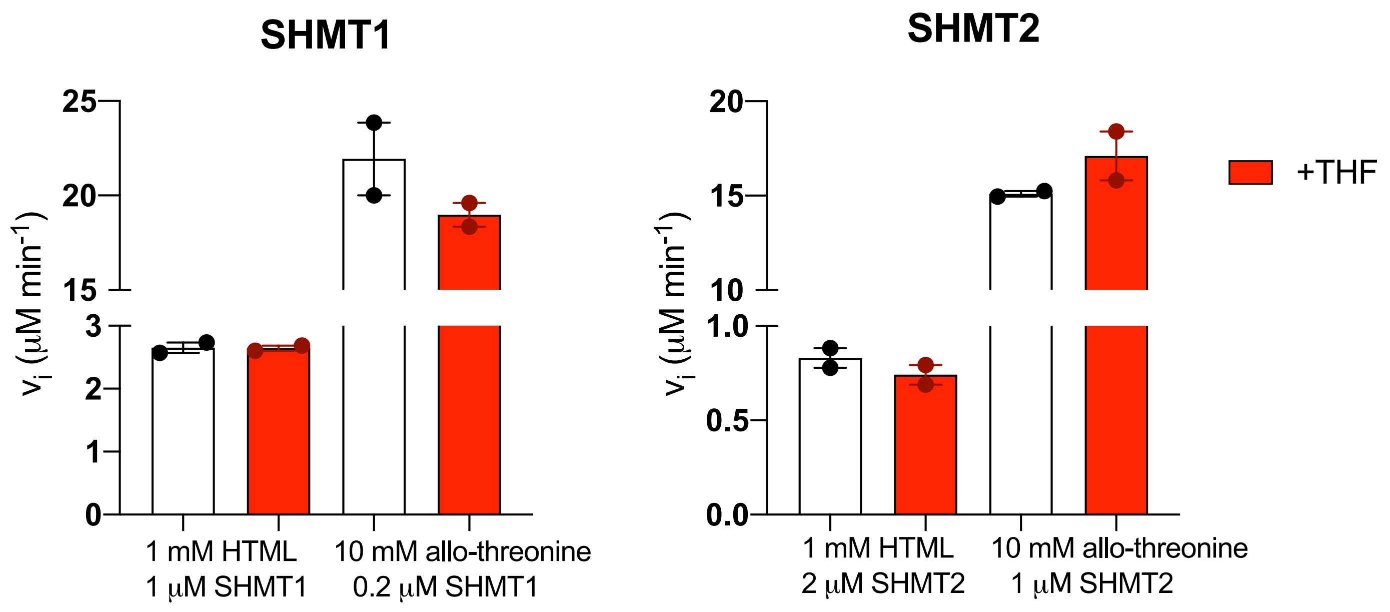

Supplementary Figure 16: Aldolase activity of human SHMTs in the presence of tetrahydrofolate. Barplot of the aldolase activity of SHMT1 (left) and SHMT2 (right) towards HTML and L-*allo*-threonine in the absence (white) or in the presence (red) of 40 μM of tetrahydrofolate (THF). Data are presented as mean values ± SEM; n=2 independent experiments. Source data are provided as a Source Data file.

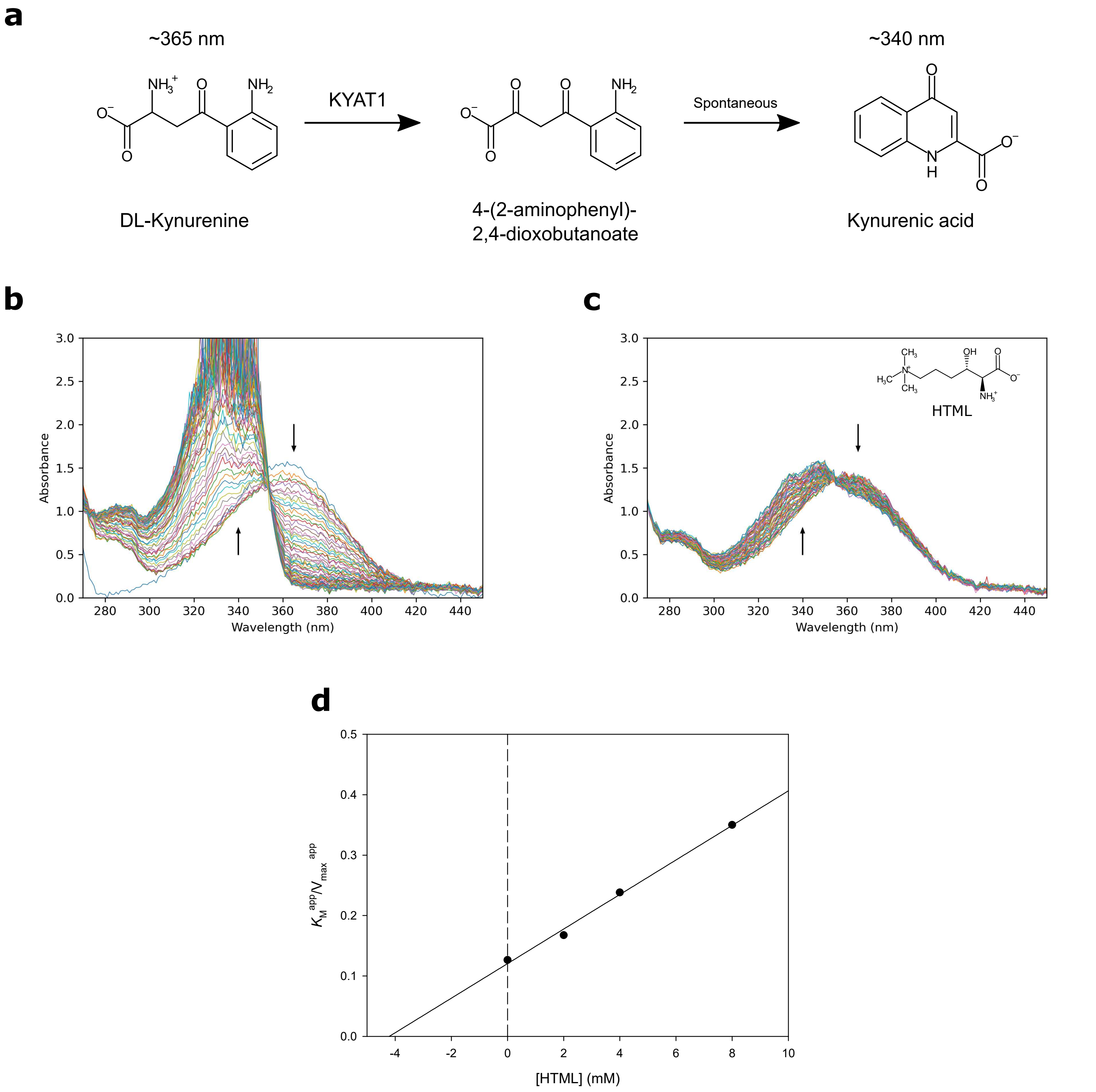

Supplementary Figure 17: Characterization of HTML as inhibitor of human KYAT 1

a, Transaminase reaction of kynurenine (365 nm) by KYAT1 and subsequent spontaneous reaction forming kynurenic acid (340 nm). **b,c** Time-resolved UV-Vis spectra showing the conversion of DL-kynurenine (0.25 mM) to kynurenic acid. Spectra were collected every 30 seconds for 30 minutes, in the absence (**b**) or in the presence (**c**) of 0.5 mM HTML. **d**, Lineweaver-Burk double-reciprocal secondary plot of the inhibition by HTML of kynurenine aminotransferase activity of KYAT1. Source data are provided as a Source Data file.

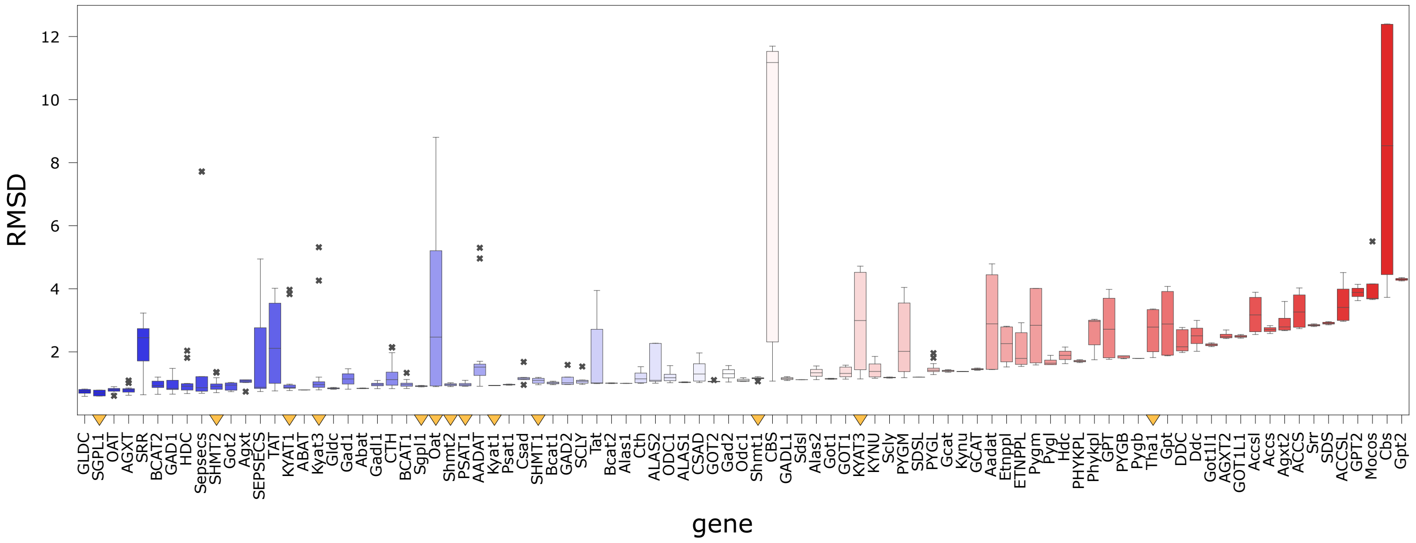

Supplementary Figure 18: RMSD between AlphaFold and SWISS-MODEL models of the enzyme set. Boxplot of Cα RMSDs calculated by the alignment between the AlphaFold models and all the corresponding templates in the SWISS-MODEL repository (SMR). The box indicates the interquartile range (IQR) divided by the median, whiskers extend to a maximum of 1.5 × IQR beyond the box. Outliers are shown as cross markers. X-axis is ordered by minimum values and colored with a blue-white-red gradient. Genes encoding proteins selected for experimental validation are highlighted with orange arrowheads on the x-axis. Source data are provided as a Source Data file.

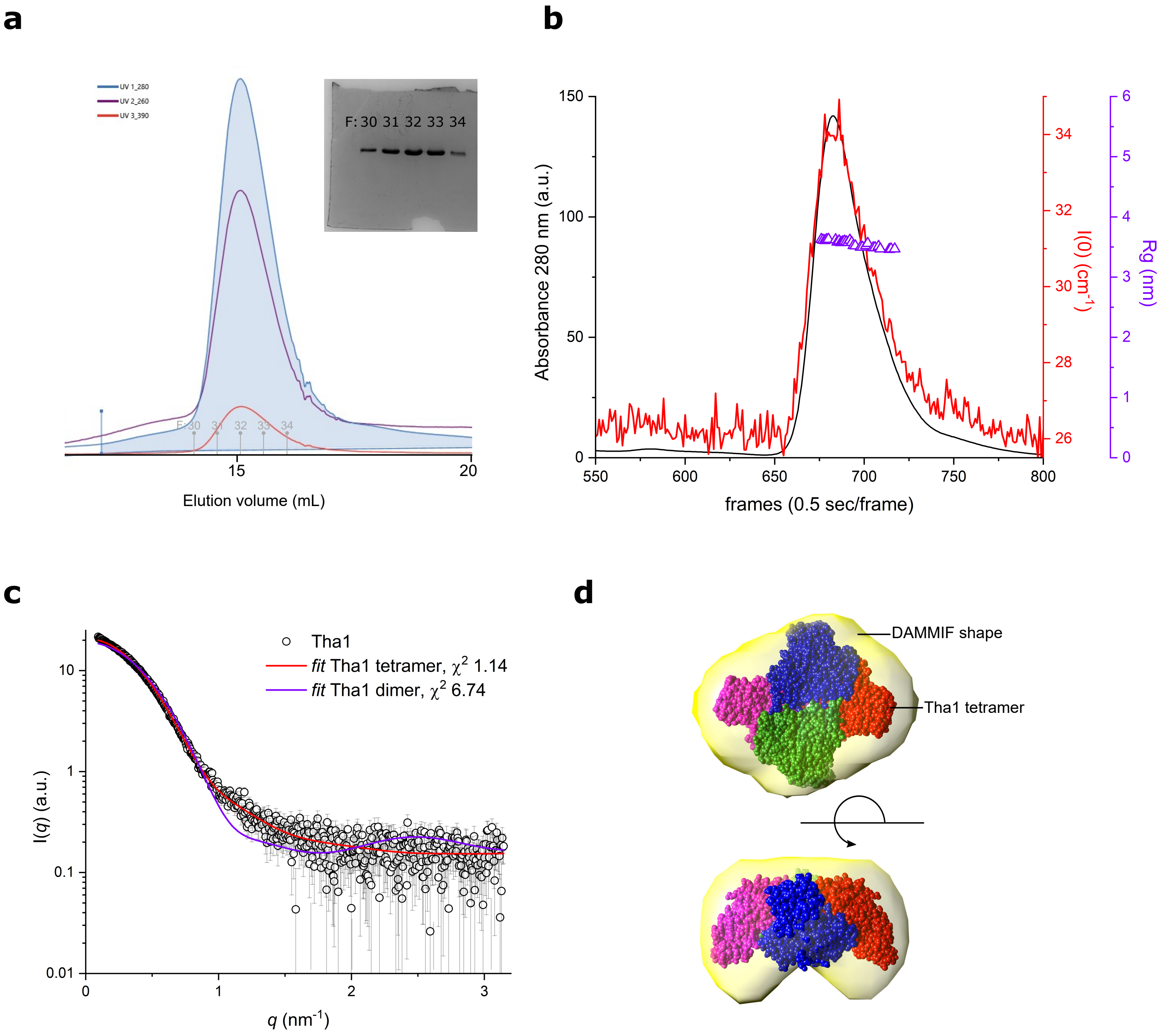

Supplementary Figure 19: Quaternary structure characterization of mouse Tha1.

a, Size exclusion chromatography (SEC) traces at different wavelengths (260, 280 and 390 nm) of Tha1/HTMLA 1 protein used for crystallization experiments. Inset shows SDS-PAGE of the protein fractions (F) corresponding to the main elution peak. **b**, SEC-SAXS chromatogram of the Tha1/HTMLA tetramer. The red and black lines represent the total summed scattering intensity and UV trace at 280 nm, respectively, for Tha1/HTMLA; blue triangles represent the calculated radius of gyration, Rg in nm, in the selected frames. The stable Rg values indicate that the elution peak is mainly composed by Tha1/HTMLA tetramer. **c**, Shown is I(q) versus q experimental SAXS profiles for Tha1/HTMLA (black circles) with CRYSOL fit using the known atomic structure of Tha1/HTMLA as tetramer (red line) or as dimer (blue line). The χ^2 values represent the discrepancy between the calculated scattering of the tetramer or dimer structures and the experimental data. Data clearly shows that Tha1/HTMLA exists in solution as tetramer. SAXS curves were obtained through averaging of buffer-background subtracted frames across the entire elution traces of the SEC-SAXS experiment (approximately n = 50 frames averaged). Error bars represent an estimate of the experimental error, σ, on the intensity recorded for each value of q assigned by data reduction software. **d**, ab initio DAMMIF model for Tha1/HTMLA superposed with the atomic structure of the tetramer. DAMMIF envelope is shown in yellow as volume model. Source data are provided as a Source Data file.

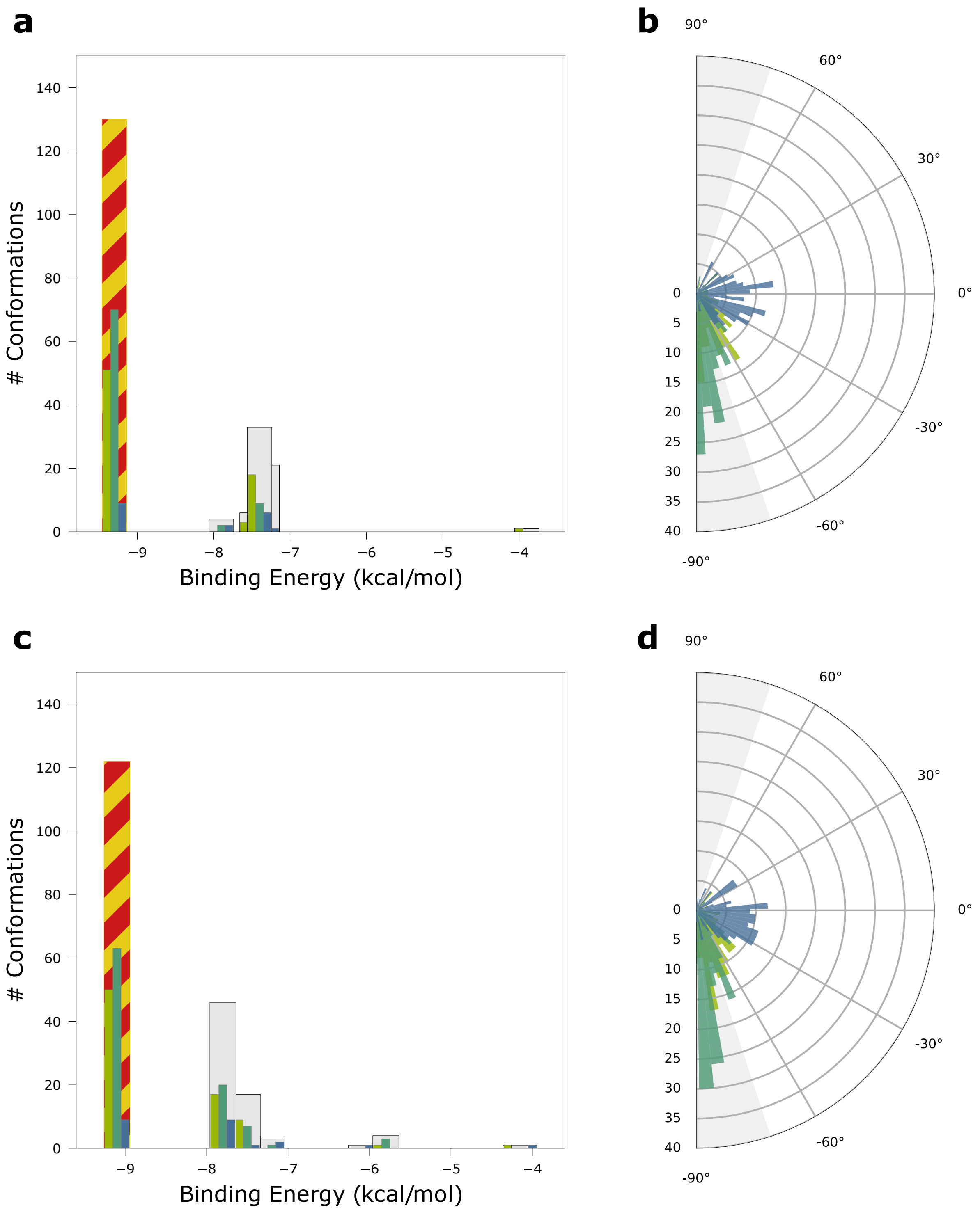

Supplementary Figure 20: Docking results with different models of Tha1.

Clustering of the HTML-PLP conformations at the Tha1 active site obtained with HTML-OSMES applied to AlphaFold model obtained by adding Tha1 crystal structure as template (**a, b**) or AlphaFold model obtained by using only Tha1 crystal structure as template (**c, d**). Bar plots show the distribution of χ_1 (blue), χ_2 (emerald) and χ_3 (kiwi) angles in each cluster, with the best and the largest cluster highlighted in red and yellow respectively. Circular plots show the cumulative distribution of the three χ angles for all clusters. $|\sin(\chi)| \geq 0.95$ values are defined by gray areas. Source data are provided as a Source Data file.

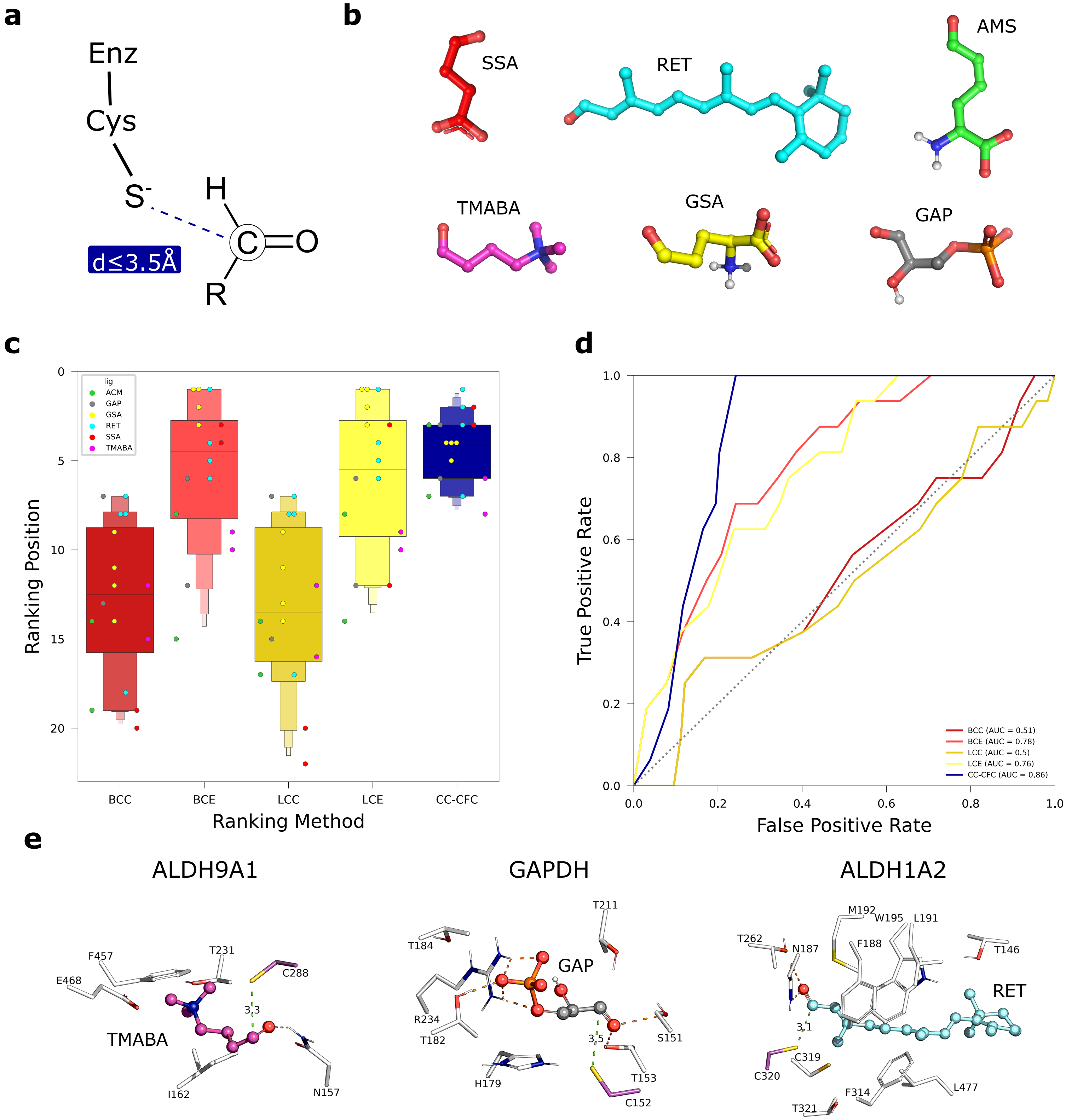

Supplementary Figure 21: Evaluation of different ranking methods of OSMES with known substrates of aldehyde dehydrogenases.

a, Expected catalytically favorable conformation of the docked aldehyde substrate. The pose is considered in Near Attack Conformation (NAC) when the distance between thiolate anion and aldehyde carbon (d) is ≤ 3.5 Å. **b**, Substrates considered in OSMES analysis: succinic semialdehyde (SSA), retinaldehyde (RET), 2-aminomuconic semialdehyde (AMS), 4-trimethylaminobutyraldehyde (TMABA), glutamate-5-semialdehyde (GSA), glyceraldehyde 3-phosphate (GAP). The energy-minimized conformation of each substrate is shown in ball-and-stick representation. Carbon atoms are colored as in panel c; non-carbon atoms are colored according to CPK. **c**, Letter-value plot showing the distribution of the validation set (n=16); the band indicates the median, the main box indicates the second and third quartiles with every further minor box splitting the remaining data into two halves. Individual dots represent ranking positions of positive controls. **d**, Receiver operating characteristic curve (ROC) for the different ranking methods colored as in panel c; the dotted diagonal represents an area under curve (AUROC) value of 0.5. **e**, Structural representation of the lowest-energy binding modes obtained by docking the substrates (TMABA, GAP, RET) in the corresponding enzymes (ALDH9A1, GAPDH, ALDH1A2). In all these cases the catalytic cluster (CC) corresponds to the best cluster (BC). Polar interactions are indicated with orange dashes. Substrates are colored as in panel b, the catalytic cysteine is colored in magenta. Source data are provided as a Source Data file.

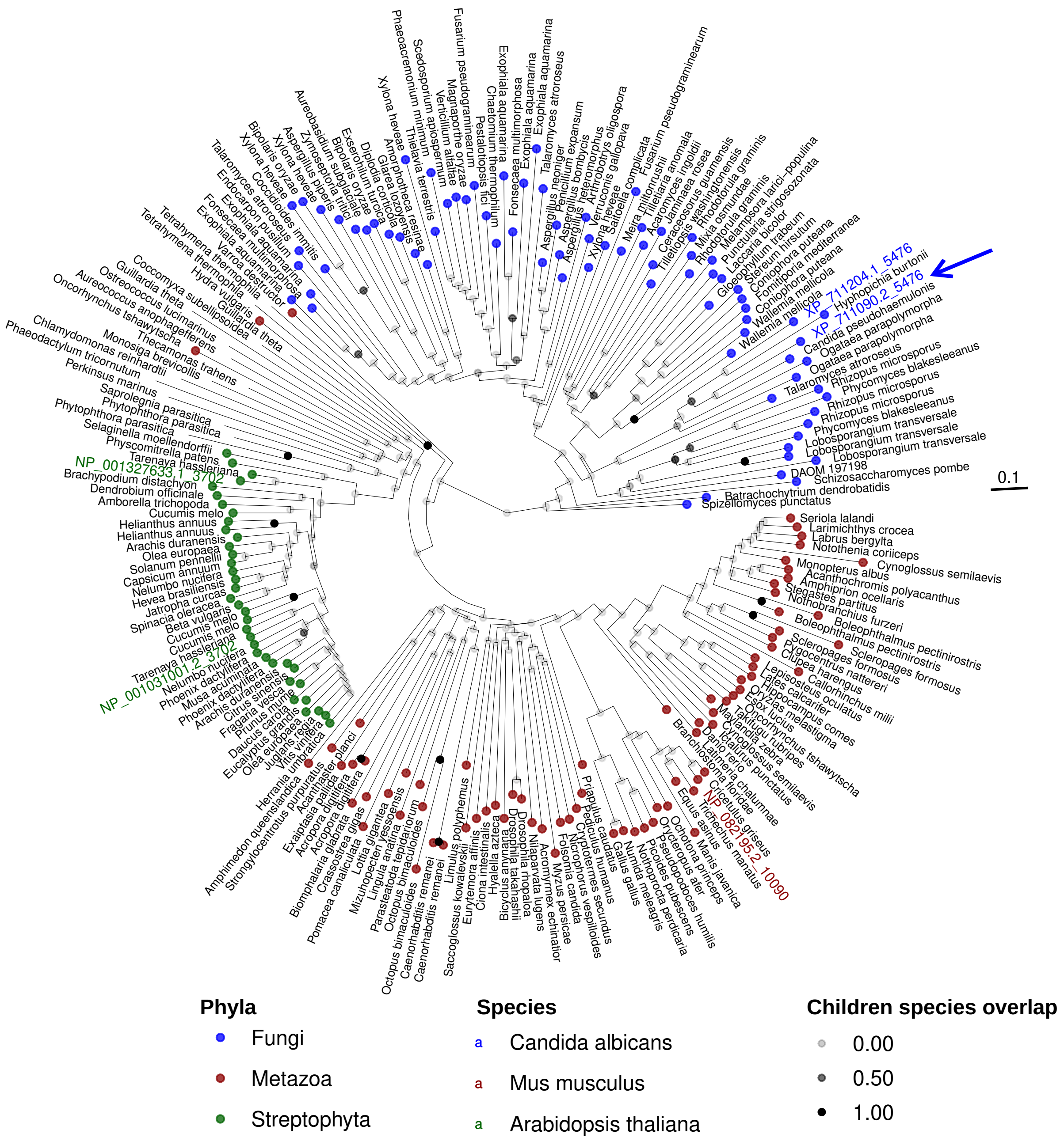

Supplementary Figure 22: Phylogeny of Tha1 in eukaryotes.

Midpoint-rooted neighbor-joining phylogenetic tree of Tha1 in representative eukaryotes. The tree shows segregation of sequences from fungi (blue tips), plants (green tips) and metazoa (red tips). The *Candida albicans* protein XP_711090 previously characterized as HTMLA[4] is indicated with an arrow. Nodes with possible gene duplication, as inferred by the proportion of overlapping species at children nodes are indicated by dark circles. Source data are provided as a Source Data file.

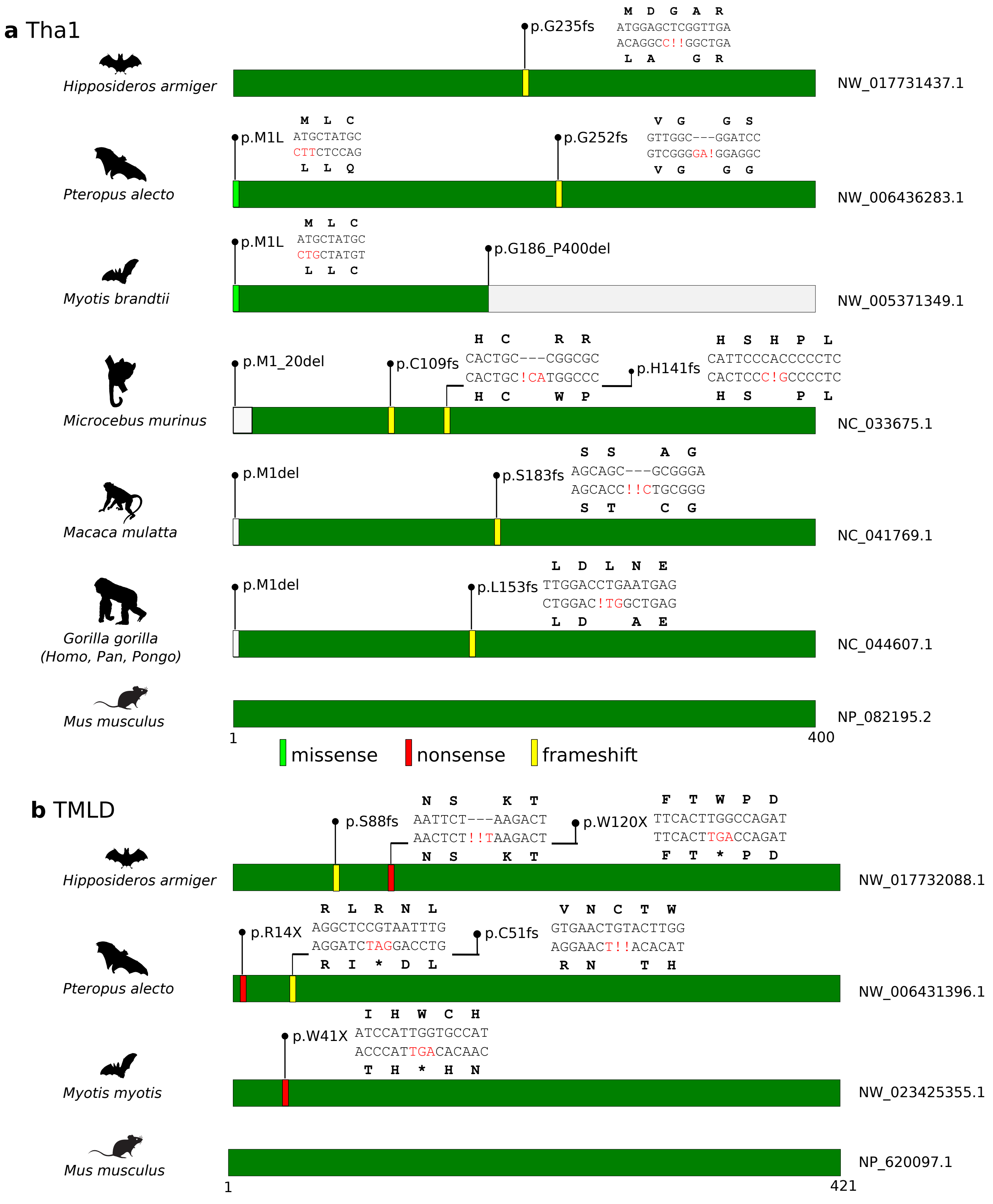

Supplementary Figure 23: Pseudogenization of Tha1 and TMLD in mammals
Examples of inactivating mutations in a) Tha1 of Chiroptera and Primates and b) TMLD of Chiroptera. Mutations disrupting the coding sequence are indicated with reference to the mouse Tha1 (NP_082195.2) and TMLD (NP_620097.1) protein sequences according to the Huret nomenclature (http://atlasgeneticsoncology.org/). Codons with disrupting mutations are colored red. Exclamation marks represent frameshift insertions/deletions. Reconstruction of pseudogene sequences was based on tblastn searches followed by GenWise analysis and Macse alignments. PhyloPic silhouettes (https://www.phylopic.org) are added to species names to aid identification.

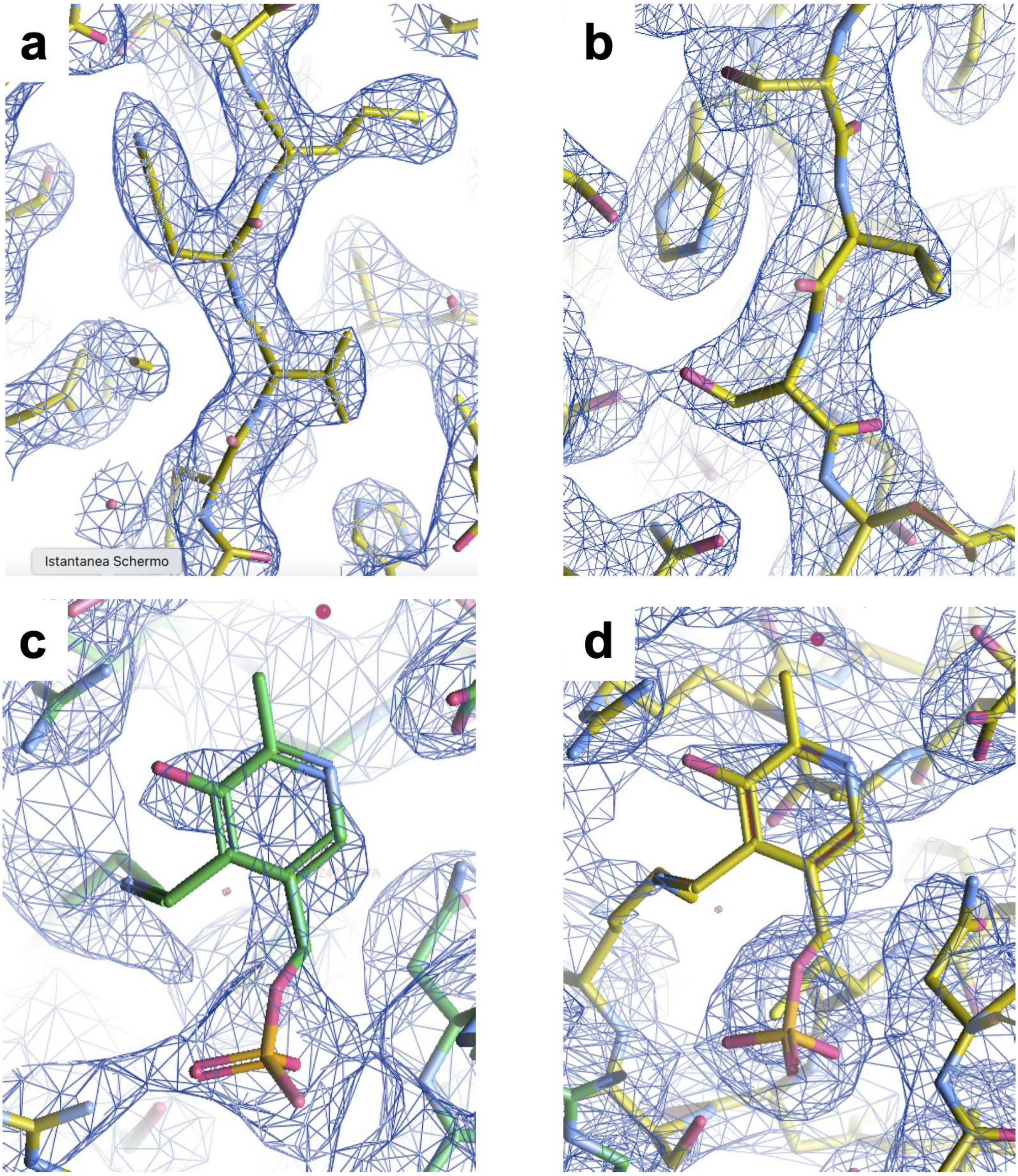

Supplementary Figure 24: Electron density maps of Tha1 crystal structures.

a and **b**, Portions of the $2F_o$-F_c electron density maps (contour level 1.0 σ) of orthorhombic (F222) and monoclinic (C2) Tha1 structures, respectively. **c** and **d**, $2F_o$-F_c SA omit-maps (contour level 0.8 σ) of PLP ligand in Tha1 monoclinic structure.

Supplementary References

1. Dembech, E. *et al.* Identification of hidden associations among eukaryotic genes through statistical analysis of coevolutionary transitions. *Proc. Natl. Acad. Sci.* **120**, e2218329120 (2023).
2. Kuznetsov, D. *et al.* OrthoDB v11: annotation of orthologs in the widest sampling of organismal diversity. *Nucleic Acids Res.* **51**, D445–D451 (2023).
3. Marchesani, F. *et al.* L-serine biosynthesis in the human central nervous system: Structure and function of phosphoserine aminotransferase. *Protein Sci.* **32**, e4609 (2023).
4. Strijbis, K. *et al.* Identification and characterization of a complete carnitine biosynthesis pathway in *Candida albicans*. *FASEB J.* **23**, 2349–2359 (2009).
